# Intracerebroventricular Injection of Alarin Increased Glucose Uptake in Skeletal Muscle of Diabetic Rats

**DOI:** 10.1371/journal.pone.0139327

**Published:** 2015-10-06

**Authors:** Zhenwen Zhang, Yongkang Wu, Shudong Sheng, Lili Guo, Biao He, Penghua Fang, Mingyi Shi, Ping Bo, Yan Zhu

**Affiliations:** 1 Department of Endocrinology, Clinical Medical College, Yangzhou University, Yangzhou, Jiangsu, China, 225001; 2 Key Laboratory of Gerontal Medicine, Medical College, Yangzhou University, Yangzhou, Jiangsu, China, 225001; 3 Department of Neurosurgery, First People's Hospital, Yangzhou University, Yangzhou, China, 225001; 4 School of Medicine, Yangzhuo Polytechnic College, Yangzhou, Jiangsu, China, 225009; Chi-Mei Medical Center, TAIWAN

## Abstract

In order to investigate the central effect of alarin on glucose uptake, we administered alarin and/ or its inhibitor, ala6-25Cys into the cerebral ventricles of the type 2 diabetic rats. Then the relative parameters about glucose uptake in skeletal muscles were measured. We found that central treatment with alarin significantly increased the food intake, body weight and glucose infusion rates in hyperinsulinemic euglycemic clamp tests of the animals. Besides, the treatment also enhanced 2-deoxy-[^3^H]-D-glucose uptake, vesicle-associated membrane protein 2 contents, glucose transporter 4 protein and mRNA expression, as well as pAkt^Thr308^, pAkt^Ser473^ and total Akt levels in muscle cells, but reduced plasma glucose and insulin levels of the rats. All of the alarin-inducing events may be antagonised by central injection of ala6-25Cys. These results suggest that central administration of alarin stimulates glucose uptake mediated by activation of Akt signal pathway in type 2 diabetic animals.

## Introduction

Alarin, a 25-amino-acid neuropeptide, is identified as the third member of the galanin family besides galanin and galaninlike peptide (GALP). Alarin was discovered as an alternate transcript of the GALP gene, excluded exon 3 from the GALP gene, in the gangliocyte of human neuroblastic tumors, and subsequently alarin mRNA was detected in the brain of rodents [1, 2]. It shares same 5 conserved amino acids at the N-terminal region as GALP [3]. High intensity alarin-like immunoreactivity was detected in accessory olfactory bulb, hypothalamus, medial preoptic area, amygdala, trigeminal complex, ventral cochlear nucleus, facial nucleus and epithelial layer of plexus choroides [4].

To date, the function of alarin in brain is scarcely known, reported only that central injection of alarin stimulated orexigenic behavior, weight gain and gonadotropin hormone secretion in male rats [1, 4, 5]. The roles of alarin seem to be mediated by unknown specific alarin receptors [6], as alarin lacks homology to galanin and is unable to compete with galanin for known galanin receptors [4]. Recently we reported that central adminisreation of alarin ameliorated insulin resistance in adipocytes of diabetic rats [7]. Because alarin can increase body weight gain of animals, in this study we further investigate whether central administration of alarin may promote glucose uptake via increasing glucose transporter 4 (GLUT4) and vesicle-associated membrane protein 2 (VAMP2) expression in skeletal muscle of the type 2 diabetic rats as well as the possible mechanisms involved.

## Materials and Methods

### Materials

Alarin was purchased from the Peptide Institute Inc, Japan. 2-Deoxy-D-[^3^H]glucose ([^3^H]2-DG) from Perkin Elmer, Boston, MA, USA. VAMP2 and GLUT4 antibodies from Santa Cruz Biotechnology Inc, USA. pAkt^Thr308^, pAkt^Ser473^ and total Akt antibodies from Cell Signaling Technology, Danvers, MA, USA. Dulbecco's modified Eagle's medium (DMEM) were from Gibco BRL, Grand Island, NE, USA. Alarin inhibitor ala6-25Cys (SSPFPPRPTRAGRETQLLRSC) was synthesized by GL Biochem, Shanghai, China.

### Animal grouping

The experiment was conducted using 150 ± 10 g Wistar male rats purchased from the Animal Center of Yangzhou University. This study and all animal protocols used in this research have been approved specifically by the Animal Care and Use Committee (ACUC) of Yangzhou University. The rats were provided with free access to a high-fat diet (59% fat, 21% protein & 20% carbohydrate) and water under a 12:12-h dark-light regime at 22 ± 2°C and 50% humidity. Eight weeks later, the rats were intraperitoneally injected with 30 mg/kg streptozocin [8]. After another two weeks animals with fast blood glucose level over 11.1 mmol/L were taken as type 2 diabetic models (T2DM). Sixty four model rats were randomly divided into four groups of 16 each: diabetic control, alarin group, ala6-25Cys group, and ala6-25Cys + alarin group. In addition, a healthy control group fed with a standard rat diet was set up (n = 16).

### Intracerebroventricular injection

After anesthetized with 50 mg/kg amobarbital sodium (i.p.), all rats were positioned in a stereotaxic frame and inserted with a 22-gauged stainless steel guide cannula into the lateral ventricle (AP), -0.8 mm; V, 3.3 mm and L, 1.4 mm as described previously [9]. After 7 day recovery, rats from alarin and ala6-25Cys group were respectively injected with 2 nmol alarin and 5 nmol ala6-25Cys in 5 μl artificial cerebrospinal fluid (in mM: 133.3 NaCl, 1.2 MgCl_2_, 0.6 NaH_2_PO_4_, 1.3 CaCl_2_, 32.0 NaHCO_3_, 3.4 KCl and 3.4 glucose, pH 7.4 by 0.5 M hydrochloric acid) through the cannula at 9:00–10:00 am once a day for one week. While rats from ala6-25Cys + alarin group with both reagents. The rats in both control groups were perfused with 5 μl artificial cerebrospinal fluid.

### ^3^H-2DG and hyperinsulinemic euglycemic clamp tests

Fasted for 24 h from the last injection, half of rats in every group (n = 8) was intraperitoneally treated with 250 mg/kg 2-deoxy-[^3^H]-D-glucose (^3^H-2DG). At 30 min after the injection, the animals were sacrificed by an overdose of Nembutal (200 mg/kg) and the carcasses were discarded according to the rules of ACUC of Yangzhou University. The 6–7 g hindlimb muscle and 4 ml artery blood were rapidly collected and stored at –80°C until later processing.

The other half of rats in every group (n = 8) were subjected to the hyperinsulinemic-euglycemic clamp tests as previously described [8]. After fasted 12 h these animals were anesthetized and catheterized in right carotid artery for blood sampling and in left jugular vein for infusion of insulin at a constant rate of 2 mU/kg min and 10% glucose at variable rates as needed to clamp blood glucose levels at 5 ± 0.5 mmol/L until the end of the test. The glucose infusion rates were calculated corresponding to the last 6 samplings at the clamp level. Once above experiments were completed, the rats were sacrificed with the aforementioned method.

### C2C12 Cell culture and ^3^H-2-DG uptake in vitro

Cell culture and glucose uptake in vitro were carried out by the method described by Shen et al. [8]. Briefly, C2C12 myoblasts were cultured in DMEM with 10% fetal bovine serum at 37°C in a 5% CO_2_ atmosphere for 16 h. After 90% confluence had been reached they were differentiated to myotubes using 2% horse serum and used for the experiments. Then the cells were washed 3 times with PBS (pH 7.4) and subsequently incubated for 30 min in the following medium: DMEM alone, DMEM containing either 1 nM, 3 nM, 10 nM and 30 nM alarin or 3 nM ala6-25Cys, or the combination of both 30 nM alarin and 3 nM ala6-25Cys. ^3^H-2DG (0.5 mM) was added to the cells, which were then incubated for 15 min. Finally, the reaction was terminated by adding ice-cold PBS, and the cells were dissolved in 1 N NaOH for measurement of ^3^H-2DG uptake by a liquid scintillation counting (Tri-Carb 2000, Packard Instrument Co.).

### Membrane subfractionation and ^3^H-2DG uptake in vivo

The membrane fractions from muscle homogenates were separated by sucrose-gradient centrifugation [8]. Briefly, 12 g of the muscle was washed, minced and homogenized in ice-cold buffer (2 mmol orthovanadate, 20 mmol NaHCO_3_, 5 mmol NaNO_3_, 250 mmol sucrose, 100 μmol PMSF, 1 μmol leupeptin, 1 μmol pepstatin A, pH 7.4). The homogenate was centrifuged at 13,000 g for 20 min at 4°C. Part of the supernatant was used for measurement of ^3^H-2DG uptake. The homogenates were centrifuged at 1200 g for 10 min (4°C). The supernatants were centrifuged at 8000 g for 10 min (4°C). Then the supernatants were layered on a 25% and 50% sucrose gradient and centrifuged at 210,000 g for 2 h (4°C) to yield the plasma membranes with L7-55Ul ultracentrifuge (Beckman, USA).

### Total RNA extraction and real-time PCR

The total RNA was isolated from 100 mg muscle tissue using the Trizol Reagent. All RNAs were reverse-transcribed to cDNA using the M-MLV reverse transcriptase. Real-time PCR was performed on an Exicycler™ 96 PCR machine (LG company, Korea) using the primer for gene amplification: GLUT4 5′-ACAGGGCAAGGATGGTAGA–3′ and 5′-TGGAGGGGAACAAGAAAGT–3′, β-actin 5′-TCCATCATGAAGTGTGACGT–3′ and 5′-GCTCAGGAGGAGCAATGAT–3′. All reactions were performed under the same conditions: 95°C for 30 min, one cycle; 95°C for 30 s, 60°C for 30 s and 72°C for 1 minute, 40 cycles. The mRNA expression levels are presented as the threshold cycle (Ct) values, normalized by the β-actin mRNA value [8].

### Western blotting

After separated on 12% SDS-polyacrylamide electrophoresis gels, the proteins from 20 mg of samples were electrophoretically transferred to polyvinylidene difluoride membranes. The membranes were respectively incubated with primary antibody against VAMP2, GLUT4, pAkt^Thr308^, pAkt^Ser473^ and total Akt respectively at 4°C overnight. Then the membranes were detected using HRP-conjugated secondary antibody. Autoradiograph was quantified using a HPIAS–2000 Image Analysis System (Championimages, China). The sum of the GLUT4 concentration in intracellular membranes and plasma membranes was taken as that in total cell membranes.

### Immunohistochemistry

Serial cross sections were cut to a thickness of 5 μm at −20°C from paraffin-embedded muscles using a Leica cryostat microtome [10]. Sections were first blocked using peroxidase blocking solution for 15 min and washed with PBS. They were incubated with the primary antibody against GLUT4 for 35 min at room temperature. After washing the sections were incubated with the secondary antibody poly-HRP for 30 min. DAB was finally applied. All sections were counterstained with hematoxylin and viewed under the light microscope.

### Statistical analysis

All values are represented as mean ± SEM. Comparisons among groups were made with ANOVA followed by Tukey’s test. Data about food intake, body weight and plasma insulin levels between before and after the experiments were compared by paired Student’s t-test. A p value<0.05 was considered significant.

## Results

### Food intake, body weight and plasma insulin levels

As shown in Table 1, the central administration of alarin increased food intake and weight gain, but reduced the plasma insulin levels in the diabetic alarin group compared with the diabetic control rats.

**Table 1:** Before and after i.c.v. administration of alarin the variation of food intake, body weight and plasma insulin level of rats.

	Food intake (g/d)		Weight (g)		Insulin (mmol/L)	
	Before	After	Before	After	Before	After
HC	14.8±1.1	15.1±1.2	294.7±11.2	301.5±13.1	4.2±0.6	4.0±0.5
DC	16.5±1.0^●^	17.3±1.8^●●^	255.8±10.2^●●^	249.9±11.4^●●^	5.7±0.9^●^	5.4±0.7^●^
Al6-25	16.2±1.2	16.9±1.5	259.8±13.2^+^	239.8±13.2^+^	5.3±0.7	6.4±0.8* ^+^
Alarin	15.6±1.8	19.2±1.7* ^++^	252.8±11.4	260.9±14.1* ^+^	5.6±0.8	4.4±0.5* ^++^
Al6-25+Al	16.1x1.8	17.5± 1.3^##^ ^△△^ ^3^	253.4±9.8	245.3±12.7^##^ ^+^	5.8±1.0	5.4±0.9^#^ ^,^ ^△^

^●^P < 0.05

^●●^P < 0.01 vs. healthy control (HC) of each division

*P < 0.05, vs. diabetic control (DC) of each division

^#^ P < 0.05

^##^P < 0.01 vs. Al6-25 group of each division

^△^P < 0.05

^△△^P < 0.01 vs. alarin group of each division

^+^P < 0.05

^++^P < 0.01 vs. each control before administration of alarin.

### Plasma glucose levels

**Fig 1:**
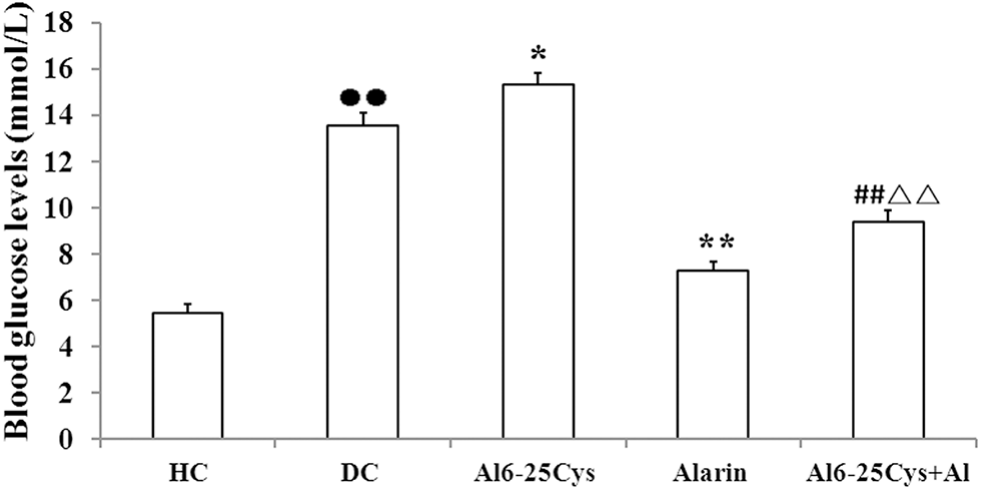
Central treatment with alarin enhanced reduced blood glucose levels in the rats (n = 8). The blood glucose levels in the alarin group were reduced compared with diabetic controls (DC). The levels in the ala6-25Cys+alarin group (ala6-25Cys+Al) was lower than ala6-25Cys group but higher than alarin group. In the diabetic control group the levels were higher than health controls (HC). The data shown are the means ± SEM. ●● P < 0.01 vs. HC; * P < 0.05, ** P < 0.01 vs. DC; ## P < 0.01 vs ala6-25Cys group; △△ P < 0.01 vs. alarin group.

The central injection of alarin for 7 days vs. vehicle reduced the plasma glucose levels in the rats. As compared with diabetic controls, the blood glucose levels were reduced by 50.5% (P < 0.01) in the alarin-treated group (P < 0.01) (Fig 1). Whereas in the ala6-25Cys + alarin group the levels were enhanced by 42.6% (P < 0.01) compared with the alarin-injected group, but reduced by 37.2% (P < 0.01) compared with the ala6-25Cys group. The levels were higher in the diabetic control group than healthy controls (P < 0.01) (S2 File).

### ^3^H-2DG uptake in vivo

**Fig 2:**
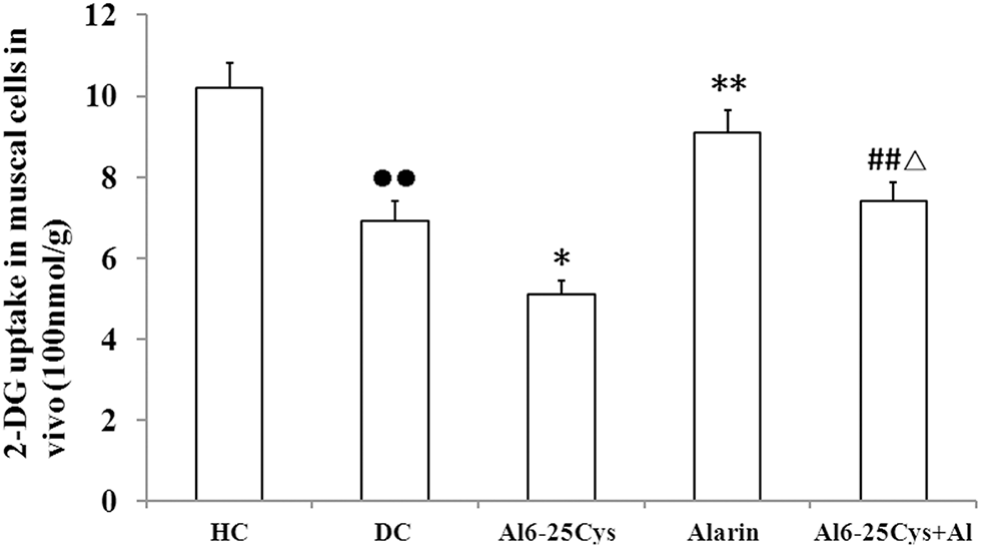
Central treatment with alarin enhanced [^3^H]-2-DG uptake in vivo (n = 8). The 2DG uptake in the alarin group was increased compared with diabetic controls (DC). The 2DG uptake in the ala6-25Cys+alarin group (ala6-25Cys+Al) was higher than the ala6-25Cys group, but lower than the alarin group. In the diabetic control group 2DG uptake was lower than health controls (HC). The data shown are the means ± SEM. ●● P < 0.01 vs. HC; * P < 0.05, ** P < 0.01 vs. DC; ## P < 0.01 vs ala6-25Cys group; △P < 0.05 vs. alarin group.

The central administration of alarin significantly stimulated glucose uptake in vivo. The 2-DG uptake was enhanced by 30.2% (P < 0.05) in the alarin-treated group as compared with diabetic controls (P < 0.01) (Fig 2). The beneficial effects of alarin were blunted by the co-administration of the alarin inhibitor, Ala6-25Cys. In the ala6-25Cys + alarin group the 2-DG uptake were decreased by 18.4% (P < 0.05) compared with the alarin-injected group, but increased 45.5% (P < 0.01) compared with the ala6-25Cys group. The 2-DG uptake was lower in the diabetic control group than healthy controls (P < 0.01) in vivo (S3 File).

### ^3^H-2DG uptake in vitro

**Fig 3:**
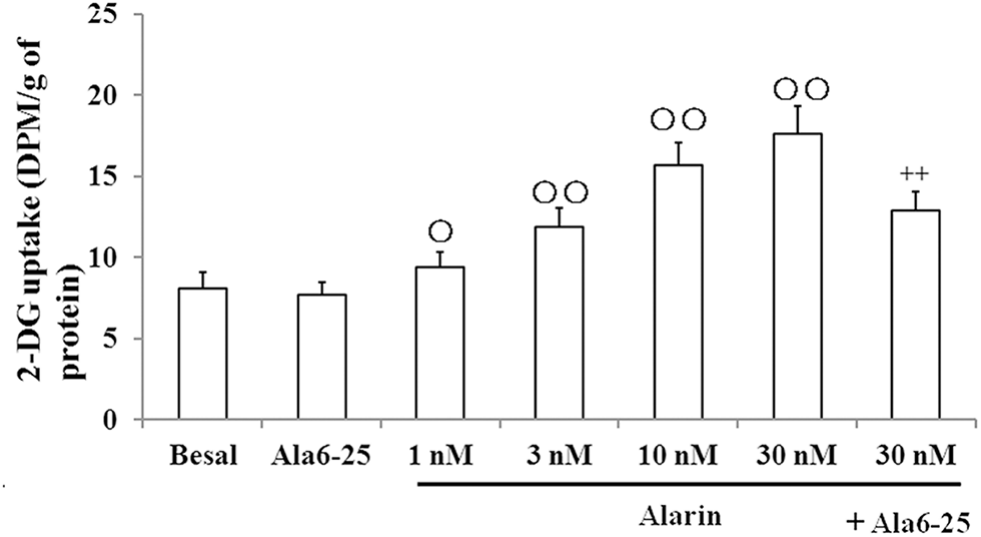
The treatment with alarin increased [^3^H]-2-DG uptake in vitro. The 2DG uptake is directly proportive to the concentration of alarin purfused in C2C12 cells. In the 30nM alarin+ ala6-25Cys group 2DG uptake was lower than the 30nM alarin group. The data shown are the means ± SEM. ○P < 0.05, ○○P < 0.01 vs. basal levels; ++ P < 0.01 vs. 30nM alarin.

We measured the 2-DG uptake in the cultured C2C12 cells in vitro. The 2-DG uptake was enhanced by 22.4% (P < 0.05), 53.9% (P < 0.01), 103.2% (P < 0.01) and 128.6% (P < 0.01) as respective perfusion with 1nM, 3 nM, 10 nM and 30 nM alarin (Fig 3). The beneficial effects of alarin were blunted by the co-administration of Ala6-25Cys. The 2-DG uptake was reduced by 26.7% (P < 0.01) in the 30 nM alarin+ ala6-25Cys group compared with the 30 nM alarin group (S4 File).

### Glucose infusion rates

**Fig 4:**
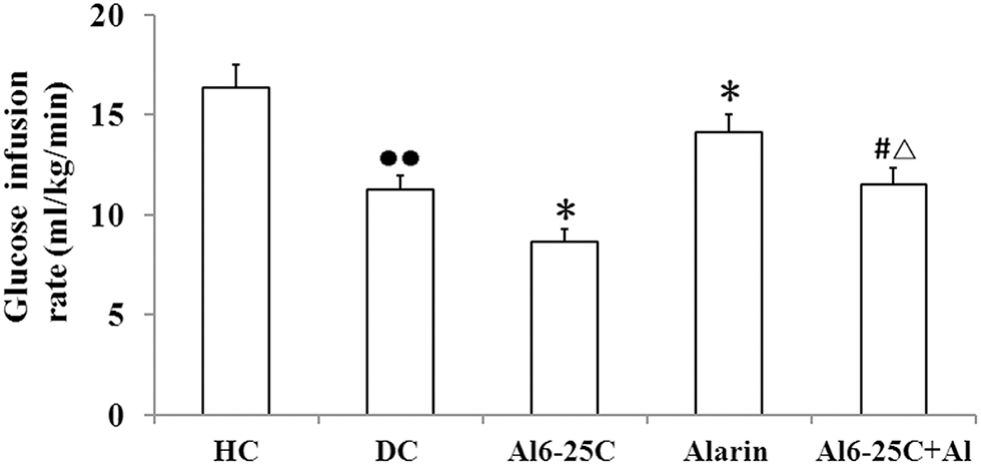
The central alarin administration significantly elevated glucose infusing rates in hyperinsulinemic-euglycemic clamp tests in the rats (n = 8). The infusing rates were higher in the alarin group than diabetic controls (DC). In the ala6-25Cys+alarin group (ala6-25Cys+Al) the rates were increased compared with the ala6-25Cys group, but attenuated compared with the alarin group. In addition, the rate in the diabetic control group was lower than healthy controls (HC). The data shown are the means ± SEM. ●● P < 0.01 vs. HC; * P < 0.05 vs. DC; # P < 0.05 vs ala6-25Cys group; △P < 0.05 vs. alarin group.

The central administration of alarin significantly elevated glucose infusing rates in the hyperinsulinemic-euglycemic clamp tests. As compared with diabetic controls, the infusion rates in the alarin group increased by 25.4% (P < 0.05), which were attenuated by co-treatment with ala6-25Cys (Fig 4). In the ala6-25Cys+alarin group the rats were enhanced by 33.3% (P < 0.05) compared with the ala6-25Cys group, but decreased by 18.5% (P < 0.05) compared with the alarin group. As same, the infusion rates were significantly lower (P < 0.01) in the diabetic control group than healthy controls (S5 File).

### GLUT4 contents in membranes of the muscles

**Fig 5:**
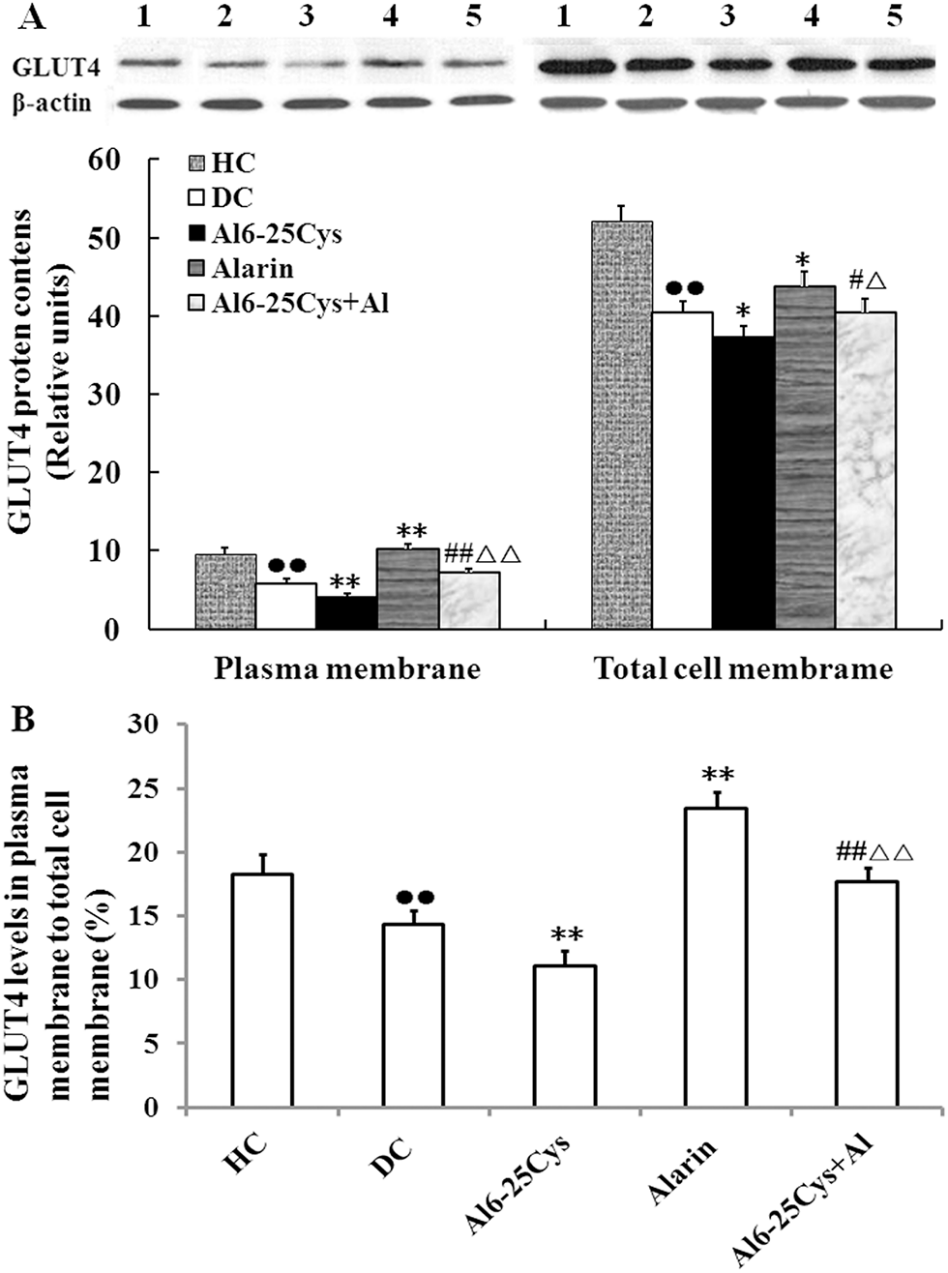
Central injection of alarin into the rats increased GLUT4 expression and trafficking in the skeletal muscles (n = 8). (A)Compared with each diabetic controls (DC) the GLUT4 levels in plasma membranes and total cell membranes of alarin groups were significantly enhanced. The immunoreactivity of both membranes in the ala6-25Cys+alarin group (ala6-25Cys+Al) was lower than each alarin group, but higher than each ala6-25Cys group. The GLUT4 expression levels in the both membranes were lower in the diabetic control group than each health controls (HC). 1 presents the health control group, 2 the diabetic control group, 3 the ala6-25Cys group, 4 the alarin group, 5 the ala6-25Cys+alarin group. (B) The radio of GLUT4 densities in plasma membranes to total cell membranes was higher in the alarin group than diabetic controls. But the ratio was lower in the ala6-25Cys + alarin group than the alarin group. The data shown are the means ± SEM. ●● P < 0.01 vs. HC; * P < 0.05, ** P < 0.01 vs. DC; # P < 0.05, ## P < 0.01 vs ala6-25Cys group; △P < 0.05, △△ P < 0.01 vs. alarin group.

We evaluated the effects of central alarin on the GLUT4 expression and translocation in skeletal muscle via the Western blot assay. As compared with diabetic controls, central treatment with alarin significantly increased the GLUT4 levels by 76.8% (P < 0.01) in plasma membranes and 8.3% (P < 0.05) in total cell membranes, which were blocked by co-injection of ala6-25Cys (Fig 5). The GLUT4 protein expression in the ala6-25Cys + alarin group in comparison to the alarin group was reduced by 30.2% (P < 0.01) in plasma membranes and 7.5% (P < 0.05) in total cell membranes, but in comparison to the ala6-25Cys group elevated by 73.9% (P < 0.01) and 8.4% (P < 0.05). The GLUT4 levels in both membranes of the diabetic controls were significantly lower than that of each healthy control (P < 0.01). Moreover, central treatment with alarin increased the ratio of GLUT4 densities in plasma membranes to total cell membranes by 63.6% (P < 0.01) in the alarin group compared with diabetic controls, suggesting that alarin boosted GLUT4 translocation to plasma membranes. But ala6-25Cys reduced the ratio by 24.4% (P < 0.01) in the ala6-25Cys + alarin group compared with the alarin group (S6 File).

**Fig 6:**
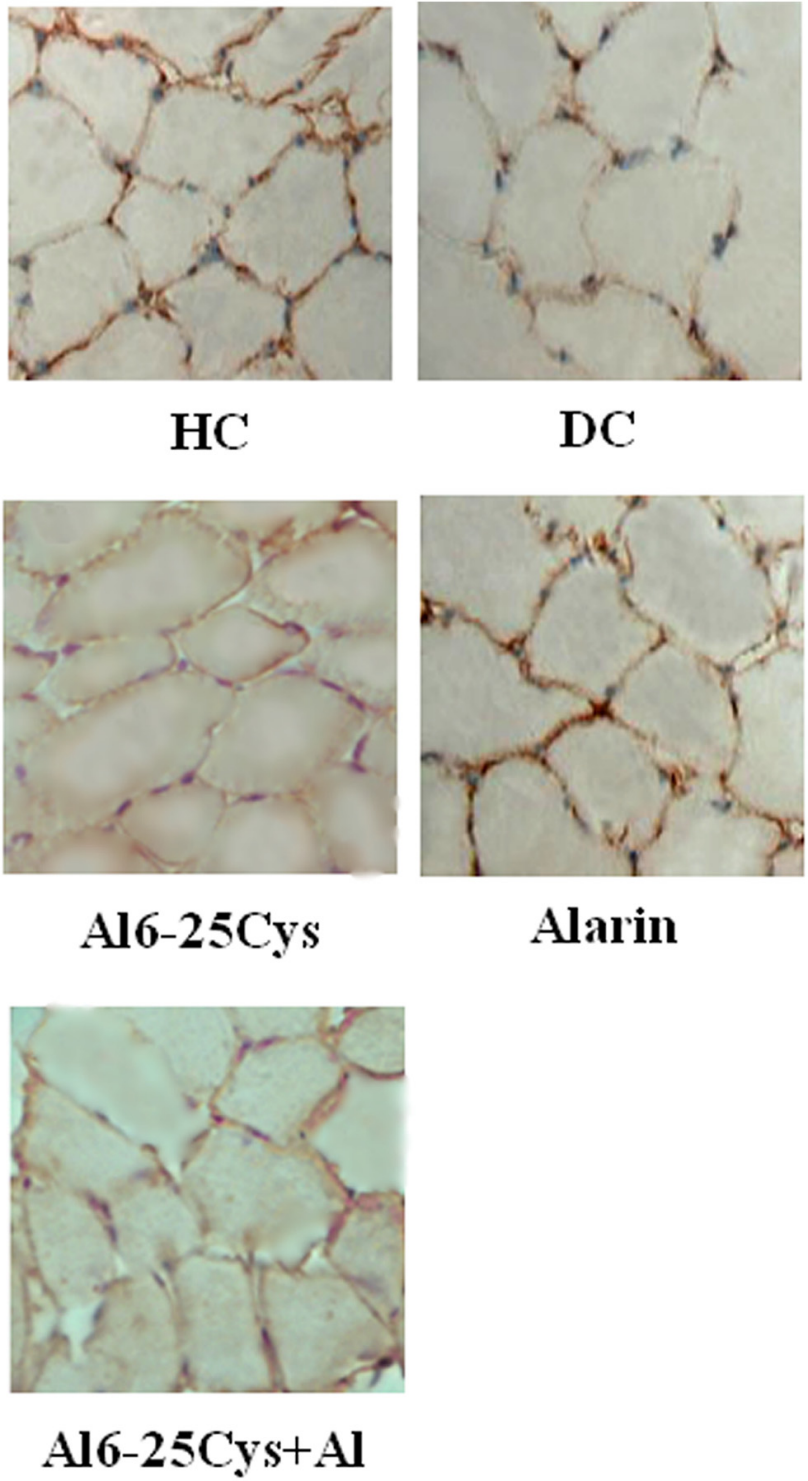
The representative photomicrographs of GLUT4-immunoreactive muscle cells following i.c.v. injection of vehicle, alarin and/or alarin antagonist Al6-25Cys (×200). The GLUT4 abundance in the plasma membrane of muscle cells was higher in the alarin group than diabetic controls (DC), and in the ala6-25Cys+alarin group (ala6-25Cys+Al) than the ala6-25Cys group. But the immunoreactivity was lower in ala6-25Cys+Al than the alarin group, and in DC than health controls (HC).

The photomicrographs of GLUT4-immunoreactive muscle cells showed that **t**he GLUT4 abundance in the plasma membrane was higher in the alarin group than diabetic controls (Fig 6), and in the ala6-25Cys+alarin group than the ala6-25Cys group. But the immunoreactivity was lower in the ala6-25Cys+alarin group than the alarin group, and in the diabetic control group than health controls.

### VAMP2 and GLUT4 mRNA expression levels

**Fig 7:**
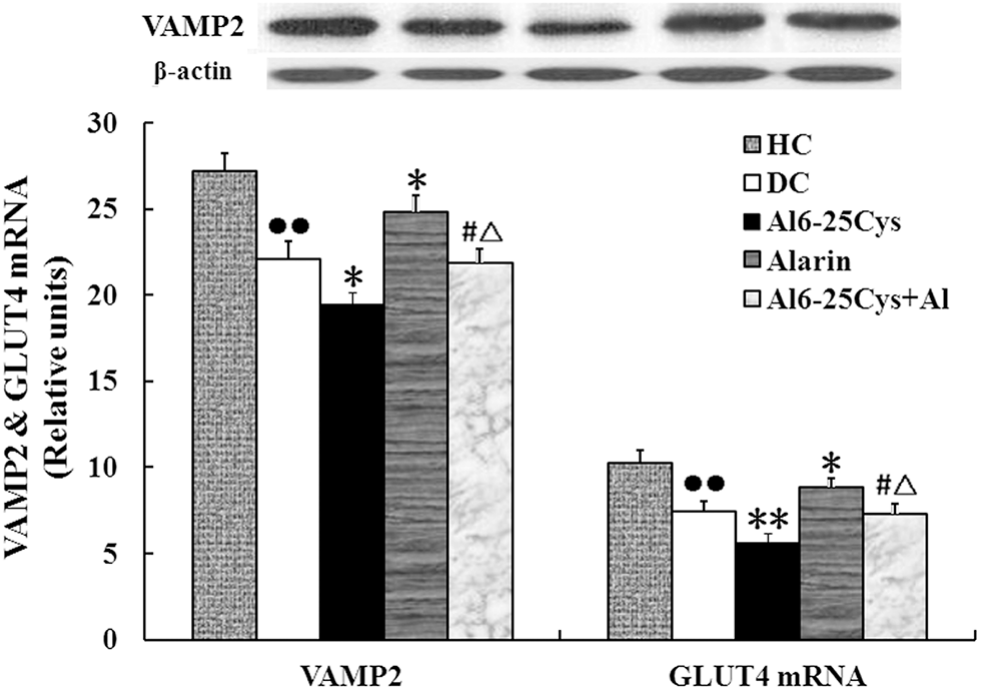
After central alarin administration the VAMP2 and GLUT4 mRNA expression levels were increased in the muscle cells of the rats (n = 8). In comparison to diabetic controls (DC), VAMP2 contents measured using western-blotting assay and GLUT4 mRNA expression levels using real time PCR in alarin groups were significantly enhanced. Both indexes in ala6-25Cys+alarin groups (ala6-25Cys+Al) were higher than the ala6-25Cys group, but lower than the alarin group. In the diabetic control group both indexes were lower than that in the health control group (HC). 1 presents the health control group, 2 the diabetic control group, 3 the ala6-25Cys group, 4 the alarin group, 5 the ala6-25Cys+alarin group. The data shown are the means ± SEM. ●● P < 0.01 vs. HC; * P < 0.05, ** P < 0.01 vs. DC; # P < 0.05 vs ala6-25Cys group; △P < 0.05 vs. alarin group.

In the current study, **w**e measured the VAMP2 levels in plasma membranes using western-blotting assay and GLUT4 mRNA expression in the muscles using real-time PCR (Fig 7). Compared with diabetic controls, central injection of alarin significantly augmented VAMP2 and GLUT4 mRNA expression by 12.4% (P < 0.05) and 19.3% (P < 0.05) respectively. VAMP2 and GLUT4 mRNA expression levels were decreased by 12.2% (P < 0.05) and 17.5% (P < 0.05) in the ala6-25Cys + alarin group compared with the alarin-treated group, but increased by 12.2% (P < 0.05) and 28.9% (P < 0.05) as compared with the ala6-25Cys-treated group. Again, the both indexes in the diabetic control group were significantly lower than that in the healthy control group (P < 0.01) (S7 File).

### pAkt levels

**Fig 8:**
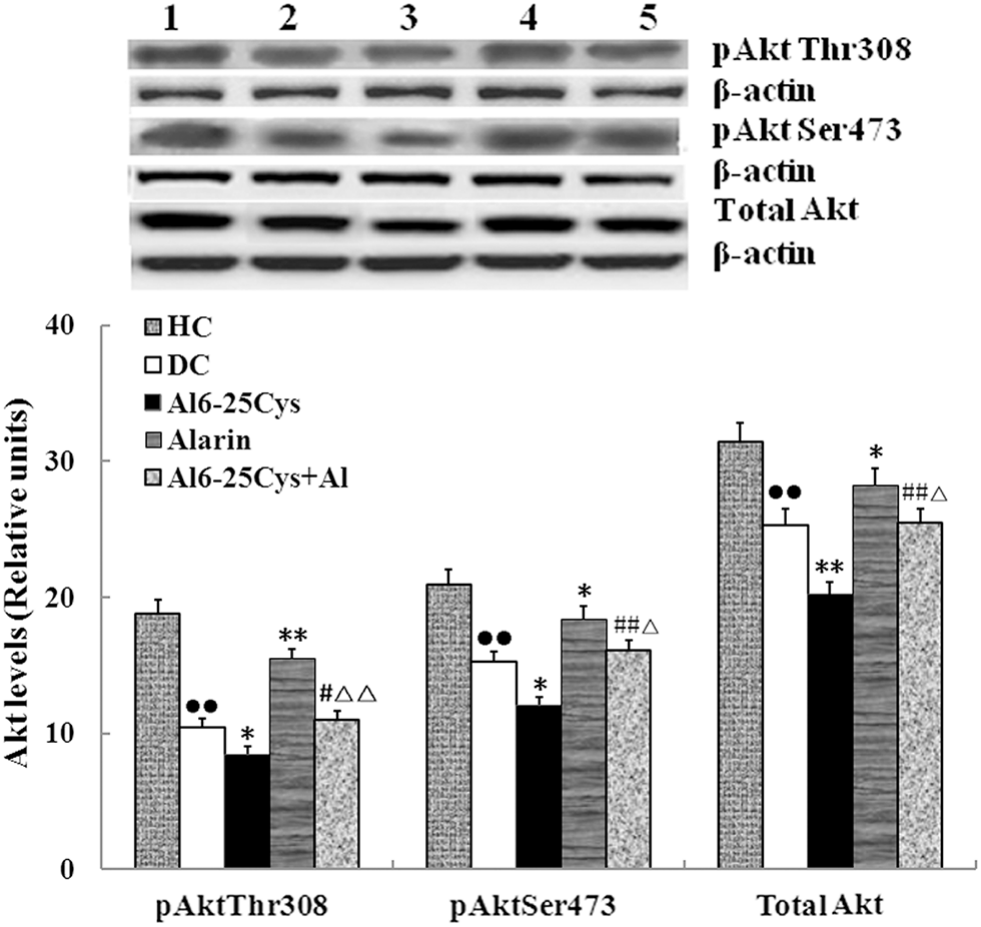
Central alarin administration increased pAkt^Thr308^, pAkt^Ser473^ and total Akt levels in the skeletal muscles of the rats (n = 8). The Akt^Thr308^,and Akt^Ser473^ phosphorylation **as well as total Akt level** were higher in the alarin group than each diabetic control (DC). Three levels in the ala6-25Cys+alarin group (ala6-25Cys+Al) were higher than the ala6-25Cys group, but lower than the alarin group. Three parameters in the diabetic control group were lower than health controls (HC). 1 presents the health control group, 2 the diabetic control group, 3 the ala6-25Cys group, 4 the alarin group, 5 the ala6-25Cys+alarin group. The data shown are the means ± SEM. ●● P < 0.01 vs. HC; * P < 0.05, ** P < 0.01 vs. DC; # P < 0.05 vs ala6-25Cys group; △P < 0.05, △△ P < 0.01 vs. alarin group.

In order to determine the signaling pathway of alarin, we measured pAkt^Thr308^, pAkt^Ser473^ and total Akt levels in the muscles [11]. As shown in Fig 8, central injection of alarin significantly elevated pAkt^Thr308^, pAkt^Ser473^ and total Akt levels by 47.6% (P < 0.01), 23.6% (P < 0.05) and 12.3% (P < 0.05) compared with each diabetic control in the skeletal muscles. The three indexes in the ala6-25Cys + alarin group were respectively attenuated by 33.5% (P < 0.01), 16.4% (P < 0.05) and 10.1% (P < 0.05) compared with the alarin group, but enhanced by 21.2% (P < 0.05), 30.6% (P < 0.01) and 26.2% (P < 0.01) compared with the ala6-25Cys group. As well, the three parameters in the diabetic control group were lower than healthy controls (P < 0.01) (S8 File).

## Discussion

Skeletal muscle, constituting about 40% of mammalian body mass, serves as a major target organ for glucose uptake from circulation [8] About 80% of the postprandial glucose uptake was achieved by skeletal muscle [12]. Impaired glucose intake in skeletal muscle is a key feature of insulin resistance and type 2 diabetes. In the present study, we surveyed several indexes of glucose uptake, including ^3^H-2DG contents, glucose infusion rates, GLUT4 protein and mRNA levels as well as VAMP2 concentration in the muscle cells to judge if central administration of alarin may increase glucose uptake in skeletal muscles of type 2 diabetic rats.

First, as a glucose analogue, 2-DG may be transported into muscles via same transporters as glucose. After taken up into cells, 2-DG is phosphorylated to 2-DG-6-phosphate, which prevents it from being released from cells again and metabolized further as its lack of a necessary 2-hydroxyl group for glycolysis. Accordingly, the 2-DG level in the cells becomes a surrogate of glucose uptake and insulin sensitivity [13].

Second, the hyperinsulinemic euglycemic clamp test is a good method to assess the capacity of glucose intake. During the clamp test, a constant insulin infusion increases the plasma glucose uptake into insulin-sensitive tissues and inhibits endogenous glucose production by liver. The decline in the plasma glucose level is prevented by a concomitantly variable rate of glucose infusion to maintain blood glucose at a clamp level. Then the amount of exogenous glucose is equal to the sum of glucose consumed by insulin-sensitive tissues, mainly by skeletal muscle. Therefore, an elevation of the glucose infusion speed suggests an increase in glucose uptake of skeletal muscles [14].

Third, GLUT4 is an important member in the glucose transporter family in charge of the insulin-stimulating glucose uptake to keep blood glucose homeostasis [15]. Only at the cell surface can GLUT4 transport glucose into cells. Increased GLUT4 translocation from intracellular storage organelles to plasma membranes is directly proportional to the enhanced amount of glucose uptake in cells [8]. The greater the GLUT4 protein at the cell surface, the higher the glucose uptake is observed.

Fourth, the GLUT4 mRNA level more likely reflected the change in GLUT4 synthesis rate rather than its half-life [14]. Therefore, the GLUT4 mRNA level may represent the quantity of GLUT4 synthesis in skeletal muscles.

Last, VAMP2 is a key protein to regulate physical fusion of trafficked GLUT4-vesicles with target membrane compartments [16]. An administration of VAMP2 neurotoxins or overexpression of dominant-interfering VAMP2 peptides inhibited GLUT4 translocation. The VAMP2 contents in plasma membranes of skeletal muscles are a biomarker of the GLUT4 translocation event.

In the current study we found that central perfusion of alarin increased ^3^H-2DG influx, glucose infusion rate in the hyperinsulinemic euglycemic clamp test, GLUT4 mRNA expression, GLUT4 and VAMP2 contents at the plasma membranes of the muscle cells.

These alarin-inducing effects may be blocked by co-treatment of alarin antagonist ala6-25Cys (SSPFPPRPTRAGRETQLLRSC), which is eliminated the first 5 amino acids from alarin (APAHRSSPFPPRPTRAGRETQLLRS). Ala6-25Cys can antagonize the promoting effects of alarin on food intake and luteinizing hormone secretion [17, 18]. These results suggest that central administration of alarin enhances glucose uptake of the skeletal muscles. In addition, perfusion of alarin played a same beneficial effect on glucose uptake in vitro, indicating that this was a direct effect of alarin on glucose uptake in the cultured myocytes.

Our previous studies found that intracerebroventricular injection of alarin significantly increased the body weight of animals, the 2DG uptake, the plasma adiponectin levels, the glucose infusion rates in hyperinsulinemic euglycemic clamp tests, the vesicle-associated membrane protein 2 as well as GLUT4 protein and mRNA levels, the ratios of GLUT4 contents in plasma membranes to total cell membranes in adipocytes, but reduced blood glucose and plasma retinol-binding protein 4 levels [7]. By comparison of our current and previous results, a conclusion can be drawn that the effects of central alarin on glucose uptake and insulin sensitivity are similar in skeletal muscles as in adipose tissues.

Akt is a key signaling molecule in the cascade pathway to trigger GLUT4 translocation after phosphorylation of Akt^Thr308^ and Akt^Ser473^ [11, 19]. The action of alarin on glucose uptake is dependent on the Akt phosphorylative activity [20]. In order to assess the impacts of central alarin signaling system on glucose uptake, we surveyed the Akt phosphorylation levels in the skeletal muscles. We observed that central injection of alarin enhanced glucose uptake concomitant with an increase in pAkt^Thr308^, pAkt^Ser473^ and total Akt levels. Co-treatment with ala6-25Cys eliminated not only alarin-induced glucose uptake but also Akt phosphorylation. Therefore, the current results suggest that central alarin stimulates glucose uptake via activation of Akt-VAMP2-GLUT4 pathway in the skeletal muscle. In addition, we found that the parameters of insulin resistance, including 2-DG uptake, glucose infusion rate, VAMP2 contents, GLUT4 protein and mRNA expression levels in the ala6-25Cys group were significantly lower than diabetic controls, suggesting there are synthesis and secretion of endogenous alarin in the central nervous system.

Date showed that central alarin can promote food intake, luteinizing hormone and gonadotropin-releasing hormone secretion via regulating the hypothalamo–pituitary–gonadal axis activity in rats [3–5]. Here, our experimental results revealed that central alarin enhanced glucose uptake in skeletal muscles of type 2 diabetic rats. Therefore, it may be speculated that the central alarin regulates glucose uptake in muscles at least in part via regulation of hypothalamo–pituitary–gonadal axis activity. Moreover, central administration of alarin increased Fos expression in hypothalamus, diencephalon and rhombencephalon [3, 4], suggesting that alarin in brain may regulate release of many central transmitters, liking NPY [3], to affect food intake and glucose uptake of subjects. The precise mechanism underlying the effect of central alarin on glucose uptake remains to be elucidated in the future.

In short, the present results indicated that central administration of alarin enhanced food intake and body weight of animals, 2-DG uptake, infusion rates in hyperinsulinemic euglycemic clamp tests, GLUT4 and VAMP2 as well as GLUT4 mRNA expression, GLUT4 translocations and Akt phosphorylation, but reduced blood glucose and insulin levels. These beneficial effects of alarin were blunted by co-administration of Ala6-25Cys. These findings suggest that Akt phosphorylation and Akt-VAMP2-GLUT4 pathway are necessary for the beneficial effect of central alarin on glucose uptake in the skeletal muscles. These extend our understanding of central effects of the alarin projective system on glucose uptake in skeletal muscles.

## Supporting Information

S1 File The effect of alarin on food intake, body weight and plasma insulin level of rats. 1.1. Food intake (Before) 1.1.1. Data 1.1.1. Statistical analysis 1.2. Food intake (After) 1.2.1. Data 1.2.2. Statistical analysis 1.2.3. Statistical analysis (Paired Samples Test) Food intake (Before vs. After) 1.3. Weight (Before) 1.3.1. Data 1.3.2. Statistical analysis 1.4. Weight (After) 1.4.1. Data 1.4.2. Statistical analysis 1.4.3. Statistical analysis (Paired Samples Test) Weight (Before vs. After) 1.5. Insulin (Before) 1.5.1. Data 1.5.2. Statistical analysis 1.6. Insulin ((After) 1.6.1. Data 1.6.2. Statistical analysis 1.6.3. Statistical analysis (Paired Samples Test) Insulin ((Before vs. After)
(DOCX)

S2 File The effect of alarin on plasma glucose levels. 1.7. Data 1.8. Statistical analysis
(DOCX)

S3 File The effect of alarin on 3H-2DG uptake in vivo. 3.1. Data 3.2. Statistical analysis
(DOCX)

S4 File The effect of alarin on ^3^H-2DG uptake in vitro. 1.9. Data 1.10. Statistical analysis
(DOCX)

S5 File The effect of alarin on glucose infusion rates. 1.11. Data 1.12. Statistical analysis
(DOCX)

S6 File The effect of alarin on GLUT4 contents in membranes of the muscles. 6.1. GLUT4 contents in plasma membranes 6.1.1. Data 6.1.2. Statistical analysis 6.2. GLUT4 contents in total cell membranes 6.2.1. Data 6.2.2. Statistical analysis 6.3. GLUT4 contents in plasma membranes to total cell membranes 6.3.1. Data 6.3.2. Statistical analysis
(DOCX)

S7 File The effect of alarin on VAMP2 and GLUT4 mRNA expression levels. 7.1. VAMP2 expression levels 7.1.1. Data 7.1.2. Statistical analysis 7.2. GLUT4 mRNA expression levels 6.2.1. Data 6.2.2. Statistical analysis
(DOCX)

S8 File The effect of alarin on Akt levels. 8.1. pAkt^Thr308^ levels 8.1.1. Data 8.1.2. Statistical analysis 8.2. pAkt^Ser473^ levels 8.2.1. Data 8.2.2. Statistical analysis 8.3. Total Akt levels 8.3.1. Data 8.3.2. Statistical analysis
(DOCX)

## Abbreviations

GALP: galaninlike peptide
GLUT4: glucose transporter 4
[^3^H]2-DG: 2-Deoxy-D-[^3^H]glucose
T2DM: type 2 diabetic models
VAMP2: vesicle-associated membrane protein 2
