# Supplementary material for: Intracerebroventricular Injection of Alarin Increased Glucose Uptake in Skeletal Muscle of Diabetic Rats
Source: PLoS One. 2015 Oct 6;10(10):e0139327. doi: 10.1371/journal.pone.0139327 (PMC4595443; doi:10.1371/journal.pone.0139327)
Supplement: S1 File — 1.1. Food intake (Before) 1.1.1. Data 1.1.1. Statistical analysis 1.2. Food intake (After) 1.2.1. Data 1.2.2. Statistical analysis 1.2.3. Statistical analysis (Paired Samples Test) Food intake (Before vs. After) 1.3. Weight (Before) 1.3.1. Data 1.3.2. Statistical analysis 1.4. Weight (After) 1.4.1. Data 1.4.2. Statistical analysis 1.4.3. Statistical analysis (Paired Samples Test) Weight (Before vs. After) 1.5. Insulin (Before) 1.5.1. Data 1.5.2. Statistical analysis 1.6. Insulin ((After) 1.6.1. Data 1.6.2. Statistical analysis 1.6.3. Statistical analysis (Paired Samples Test) Insulin ((Before vs. After) (DOCX) [file pone.0139327.s001.docx]

**1. Before and after i.c.v. administration of alarin the variation of food intake, body weight and plasma insulin level of rats**.

Table 1

_________________________________________________________________________

Food intake（g/d） Weight (g) Insulin (mmol/L)

Before After Before After Before After

_________________________________________________________________________

HC 14.8±1.1 15.1±1.2 294.7±11.2 301.5±13.1 4.2±0.6 4.0±0.5

DC 16.5±1.0^●^ 17.3±1.8^●●^ 255.8±10.2^●●^ 249.9±11.4^●●^  5.7±0.9^●●^ 5.4±0.7^●●^

Al6-25 16.2±1.2 16.9±1.5 248.7±10.7 239.8±13.2^++^ 5.3±0.7 6.4±0.8^*+^

Alarin 15.6±1.8 19.2±1.7^*++^ 252.8±11.4 260.9±14.1^*+^ 5.6±0.8 4.3±0.5^*++^

Al6-25+ Al 16.1±1.8 17.5± 1.3^##△△++^  253.4±9.8 245.3±12.7^##+^ 5.8±1.0 5.4±0.9^##△^

- 1. **Food intake (Before)**
     1. **Data**

HC DC Al6-25 Alarin Al6-25+ Al

15.2 17.5 16.3 15.2 16.4

14.8 16.2 14.9 15.6 14.7

14.1 18.2 17.2 15.9 15.1

13.8 17.4 15.5 14.4 17.4

16.2 16.8 16.7 17.3 15.6

15.6 15.9 14.8 15.2 14.5

14.9 15.7 18.4 16.8 17.9

13.6 14.6 15.7 14.2 17.5

**14.8 16.5 16.2 15.6 16.1**

- - 1. **Statistical analysis**

| **Multiple Comparisons** | | | | | | |
| --- | --- | --- | --- | --- | --- | --- |
|  | |  |  |  |  |  |
| (I) VAR00003 | (J) VAR00003 | Mean Difference (I-J) | Std. Error | Sig. | 95% Confidence Interval | |
|  |  |  |  |  | Lower Bound | Upper Bound |
| 1 | 2 | -1.76250^*^ | .55347 | .024 | -3.3538 | -.1712 |
|  | 3 | -1.40000 | .55347 | .107 | -2.9913 | .1913 |
|  | 4 | -1.18750 | .55347 | .224 | -2.7788 | .4038 |
|  | 5 | -1.36250 | .55347 | .123 | -2.9538 | .2288 |
| 2 | 1 | 1.76250^*^ | .55347 | .024 | .1712 | 3.3538 |
|  | 3 | .36250 | .55347 | .965 | -1.2288 | 1.9538 |
|  | 4 | .57500 | .55347 | .836 | -1.0163 | 2.1663 |
|  | 5 | .40000 | .55347 | .950 | -1.1913 | 1.9913 |
| 3 | 1 | 1.40000 | .55347 | .107 | -.1913 | 2.9913 |
|  | 2 | -.36250 | .55347 | .965 | -1.9538 | 1.2288 |
|  | 4 | .21250 | .55347 | .995 | -1.3788 | 1.8038 |
|  | 5 | .03750 | .55347 | 1.000 | -1.5538 | 1.6288 |
| 4 | 1 | 1.18750 | .55347 | .224 | -.4038 | 2.7788 |
|  | 2 | -.57500 | .55347 | .836 | -2.1663 | 1.0163 |
|  | 3 | -.21250 | .55347 | .995 | -1.8038 | 1.3788 |
|  | 5 | -.17500 | .55347 | .998 | -1.7663 | 1.4163 |
| 5 | 1 | 1.36250 | .55347 | .123 | -.2288 | 2.9538 |
|  | 2 | -.40000 | .55347 | .950 | -1.9913 | 1.1913 |
|  | 3 | -.03750 | .55347 | 1.000 | -1.6288 | 1.5538 |
|  | 4 | .17500 | .55347 | .998 | -1.4163 | 1.7663 |
| *. The mean difference is significant at the 0.05 level. | | | | | |  |

- 1. **Food intake (After)**
     1. **Data**

HC DC Al6-25 Alarin Al6-25+ Al

16.2 16.9 15.5 21.5 18.5

15.7 17.6 18.4 18.6 16.4

14.6 18.4 15.3 19.3 17.6

15.5 17.3 14.9 19.7 16.5

13.5 16.5 18.7 17.6 18.4

14.4 16.6 18.8 17.8 16.7

16.1 18.1 17.5 18.4 17.6

14.9 17.2 15.7 20.6 18.1

- 1. **17.3 16.9 19.2 17.5**
     1. **Statistical analysis**

| (I) VAR00003 | (J) VAR00003 | Mean Difference (I-J) | Std. Error | Sig. | 95% Confidence Interval | |
| --- | --- | --- | --- | --- | --- | --- |
|  |  |  |  |  | Lower Bound | Upper Bound |
| 1 | 2 | -2.21250^*^ | .56396 | .003 | -3.8339 | -.5911 |
|  | 3 | -1.73750^*^ | .56396 | .031 | -3.3589 | -.1161 |
|  | 4 | -4.57500^*^ | .56396 | .000 | -6.1964 | -2.9536 |
|  | 5 | -2.36250^*^ | .56396 | .002 | -3.9839 | -.7411 |
| 2 | 1 | 2.21250^*^ | .56396 | .003 | .5911 | 3.8339 |
|  | 3 | .47500 | .56396 | .915 | -1.1464 | 2.0964 |
|  | 4 | -2.36250^*^ | .56396 | .002 | -3.9839 | -.7411 |
|  | 5 | -.15000 | .56396 | .999 | -1.7714 | 1.4714 |
| 3 | 1 | 1.73750^*^ | .56396 | .031 | .1161 | 3.3589 |
|  | 2 | -.47500 | .56396 | .915 | -2.0964 | 1.1464 |
|  | 4 | -2.83750^*^ | .56396 | .000 | -4.4589 | -1.2161 |
|  | 5 | -.62500 | .56396 | .801 | -2.2464 | .9964 |
| 4 | 1 | 4.57500^*^ | .56396 | .000 | 2.9536 | 6.1964 |
|  | 2 | 2.36250^*^ | .56396 | .002 | .7411 | 3.9839 |
|  | 3 | 2.83750^*^ | .56396 | .000 | 1.2161 | 4.4589 |
|  | 5 | 2.21250^*^ | .56396 | .003 | .5911 | 3.8339 |
| 5 | 1 | 2.36250^*^ | .56396 | .002 | .7411 | 3.9839 |
|  | 2 | .15000 | .56396 | .999 | -1.4714 | 1.7714 |
|  | 3 | .62500 | .56396 | .801 | -.9964 | 2.2464 |
|  | 4 | -2.21250^*^ | .56396 | .003 | -3.8339 | -.5911 |

- - 1. **Statistical analysis (Paired Samples Test)**

**Food intake (Before vs. After)**

|  | | | | | | | | | | | |
| --- | --- | --- | --- | --- | --- | --- | --- | --- | --- | --- | --- |
| VAR00003 | | Paired Differences | | | | | | | t | df | Sig. (2-tailed) |
|  |  | Mean | | | Std. Deviation | Std. Error Mean | 95% Confidence Interval of the Difference | |  |  |  |
|  |  |  |  |  |  |  | Lower | Upper |  |  |  |
| 1 |  | |  | -.33750 | 1.50707 | .53283 | -1.59744 | .92244 | -.633 | 7 | .547 |
| 2 |  | |  | -.78750 | 1.22526 | .43319 | -1.81184 | .23684 | -1.818 | 7 | .112 |
| 3 |  | |  | -.67500 | 2.25373 | .79681 | -2.55916 | 1.20916 | -.847 | 7 | .425 |
| 4 |  | |  | -1.51250 | 1.01057 | .35729 | -2.35736 | -.66764 | -4.233 | 7 | .004 |
| 5 |  | |  | -3.05000 | 1.13389 | .40089 | -3.99796 | -2.10204 | -7.608 | 7 | .000 |
|  | | | | | |  |  |  |  |  |  |

- 1. **Weight (Before)**
     1. **Data**

HC DC Al6-25 Alarin Al6-25+ Al

391.6 244.1 241.6 264.1 251.6

291.4 261.5 243.2 251.5 240.3

288.1 258.7 258.7 258.7 256.7

297.6 264.3 247.6 244.3 244.3

300.5 254.9 246.5 254.9 253.9

293.2 266.7 248.1 237.6 246.5

288.4 247.6 252.2 264.9 265.4

306.7 248.3 251.7 246.7 248.3

**294.7 255.8 248.7 252.8 250.9**

- - 1. **Statistical analysis**

| **Multiple Comparisons** | | | | | | |
| --- | --- | --- | --- | --- | --- | --- |
|  | |  |  |  |  |  |
| (I) VAR00003 | (J) VAR00003 | Mean Difference (I-J) | Std. Error | Sig. | 95% Confidence Interval | |
|  |  |  |  |  | Lower Bound | Upper Bound |
| 1 | 2 | 38.92500^*^ | 3.34437 | .000 | 29.3097 | 48.5403 |
|  | 3 | 33.50000^*^ | 3.34437 | .000 | 23.8847 | 43.1153 |
|  | 4 | 41.85000^*^ | 3.34437 | .000 | 32.2347 | 51.4653 |
|  | 5 | 42.56250^*^ | 3.34437 | .000 | 32.9472 | 52.1778 |
| 2 | 1 | -38.92500^*^ | 3.34437 | .000 | -48.5403 | -29.3097 |
|  | 3 | -5.42500 | 3.34437 | .494 | -15.0403 | 4.1903 |
|  | 4 | 2.92500 | 3.34437 | .904 | -6.6903 | 12.5403 |
|  | 5 | 3.63750 | 3.34437 | .812 | -5.9778 | 13.2528 |
| 3 | 1 | -33.50000^*^ | 3.34437 | .000 | -43.1153 | -23.8847 |
|  | 2 | 5.42500 | 3.34437 | .494 | -4.1903 | 15.0403 |
|  | 4 | 8.35000 | 3.34437 | .115 | -1.2653 | 17.9653 |
|  | 5 | 9.06250 | 3.34437 | .073 | -.5528 | 18.6778 |
| 4 | 1 | -41.85000^*^ | 3.34437 | .000 | -51.4653 | -32.2347 |
|  | 2 | -2.92500 | 3.34437 | .904 | -12.5403 | 6.6903 |
|  | 3 | -8.35000 | 3.34437 | .115 | -17.9653 | 1.2653 |
|  | 5 | .71250 | 3.34437 | 1.000 | -8.9028 | 10.3278 |
| 5 | 1 | -42.56250^*^ | 3.34437 | .000 | -52.1778 | -32.9472 |
|  | 2 | -3.63750 | 3.34437 | .812 | -13.2528 | 5.9778 |
|  | 3 | -9.06250 | 3.34437 | .073 | -18.6778 | .5528 |
|  | 4 | -.71250 | 3.34437 | 1.000 | -10.3278 | 8.9028 |
| *. The mean difference is significant at the 0.05 level. | | | | | |  |

- 1. **Weight (After)**
     1. **Data**

HC DC Al6-25 Alarin Al6-25+ Al

320.1 254.2 241.7 269.6 245.3

301.7 234.8 237.4 263.9 249.5

314.1 257.7 236.8 254.4 238.4

297.6 255.4 245.4 252.5 257.3

302.3 239.6 249.6 262.3 239.5

393.2 236.2 236.2 259.5 237.4

308.4 262.3 246.5 261.2 249.6

284.7 258.9 225.1 263.5 245.1

- 1. **249.9 239.8 260.9 245.3**
     1. **Statistical analysis)**

| **Multiple Comparisons** | | | | | | |
| --- | --- | --- | --- | --- | --- | --- |
|  | |  |  |  |  |  |
| (I) VAR00003 | (J) VAR00003 | Mean Difference (I-J) | Std. Error | Sig. | 95% Confidence Interval | |
|  |  |  |  |  | Lower Bound | Upper Bound |
| 1 | 2 | 51.62500^*^ | 3.57297 | .000 | 41.3525 | 61.8975 |
|  | 3 | 61.67500^*^ | 3.57297 | .000 | 51.4025 | 71.9475 |
|  | 4 | 40.65000^*^ | 3.57297 | .000 | 30.3775 | 50.9225 |
|  | 5 | 56.25000^*^ | 3.57297 | .000 | 45.9775 | 66.5225 |
| 2 | 1 | -51.62500^*^ | 3.57297 | .000 | -61.8975 | -41.3525 |
|  | 3 | 10.05000 | 3.57297 | .058 | -.2225 | 20.3225 |
|  | 4 | -10.97500^*^ | 3.57297 | .031 | -21.2475 | -.7025 |
|  | 5 | 4.62500 | 3.57297 | .696 | -5.6475 | 14.8975 |
| 3 | 1 | -61.67500^*^ | 3.57297 | .000 | -71.9475 | -51.4025 |
|  | 2 | -10.05000 | 3.57297 | .058 | -20.3225 | .2225 |
|  | 4 | -21.02500^*^ | 3.57297 | .000 | -31.2975 | -10.7525 |
|  | 5 | -5.42500 | 3.57297 | .558 | -15.6975 | 4.8475 |
| 4 | 1 | -40.65000^*^ | 3.57297 | .000 | -50.9225 | -30.3775 |
|  | 2 | 10.97500^*^ | 3.57297 | .031 | .7025 | 21.2475 |
|  | 3 | 21.02500^*^ | 3.57297 | .000 | 10.7525 | 31.2975 |
|  | 5 | 15.60000^*^ | 3.57297 | .001 | 5.3275 | 25.8725 |
| 5 | 1 | -56.25000^*^ | 3.57297 | .000 | -66.5225 | -45.9775 |
|  | 2 | -4.62500 | 3.57297 | .696 | -14.8975 | 5.6475 |
|  | 3 | 5.42500 | 3.57297 | .558 | -4.8475 | 15.6975 |
|  | 4 | -15.60000^*^ | 3.57297 | .001 | -25.8725 | -5.3275 |
|  | | | | | |  |

- - 1. **Statistical analysis (Paired Samples Test)**

**Weight (Before vs. After)**

| **Paired Samples Test^a^** | | | | | | | | | | |
| --- | --- | --- | --- | --- | --- | --- | --- | --- | --- | --- |
| VAR00003 | | | Paired Differences | | | | | t | df | Sig. (2-tailed) |
|  |  |  | Mean | Std. Deviation | Std. Error Mean | 95% Confidence Interval of the Difference | |  |  |  |
|  |  |  |  |  |  | Lower | Upper |  |  |  |
| 1 |  |  | .22500 | .39188 | .13855 | -.10262 | .55262 | 1.624 | 7 | .148 |
| 2 |  |  | .32500 | .61354 | .21692 | -.18793 | .83793 | 1.498 | 7 | .178 |
| 3 |  |  | -1.07500 | 1.10292 | .38994 | -1.99706 | -.15294 | -2.757 | 7 | .028 |
| 4 |  |  | 1.31250 | .73764 | .26079 | .69582 | 1.92918 | 5.033 | 7 | .002 |
| 5 |  |  | .67500 | .77598 | .27435 | .02627 | 1.32373 | 2.460 | 7 | .043 |

- 1. **Insulin (Before)**

**1.5.1. Data**

HC DC Al6-25 Alarin Al6-25+ Al

3.7 6.1 5.1 5.5 5.7

4.1 4.8 4.7 5.8 5.9

4.8 5.3 5.3 5.9 5.5

3.8 5.7 5.7 5.4 5.1

4.2 6.5 4.5 4.8 5.3

3.8 4.6 4.6 4.9 5.8

4.6 6.7 6.1 5.8 6.6

4.9 6.0 6.3 6.5 6.3

- 1. **5.7 5.3 5.6 5.8**

**1.5.2. Statistical analysis**

|  | |  |  |  |  |  |
| --- | --- | --- | --- | --- | --- | --- |
| (I) VAR00003 | (J) VAR00003 | Mean Difference (I-J) | Std. Error | Sig. | 95% Confidence Interval | |
|  |  |  |  |  | Lower Bound | Upper Bound |
| 1 | 2 | -1.47500^*^ | .30079 | .000 | -2.3398 | -.6102 |
|  | 3 | -1.05000^*^ | .30079 | .011 | -1.9148 | -.1852 |
|  | 4 | -1.33750^*^ | .30079 | .001 | -2.2023 | -.4727 |
|  | 5 | -1.53750^*^ | .30079 | .000 | -2.4023 | -.6727 |
| 2 | 1 | 1.47500^*^ | .30079 | .000 | .6102 | 2.3398 |
|  | 3 | .42500 | .30079 | .624 | -.4398 | 1.2898 |
|  | 4 | .13750 | .30079 | .991 | -.7273 | 1.0023 |
|  | 5 | -.06250 | .30079 | 1.000 | -.9273 | .8023 |
| 3 | 1 | 1.05000^*^ | .30079 | .011 | .1852 | 1.9148 |
|  | 2 | -.42500 | .30079 | .624 | -1.2898 | .4398 |
|  | 4 | -.28750 | .30079 | .873 | -1.1523 | .5773 |
|  | 5 | -.48750 | .30079 | .495 | -1.3523 | .3773 |
| 4 | 1 | 1.33750^*^ | .30079 | .001 | .4727 | 2.2023 |
|  | 2 | -.13750 | .30079 | .991 | -1.0023 | .7273 |
|  | 3 | .28750 | .30079 | .873 | -.5773 | 1.1523 |
|  | 5 | -.20000 | .30079 | .963 | -1.0648 | .6648 |
| 5 | 1 | 1.53750^*^ | .30079 | .000 | .6727 | 2.4023 |
|  | 2 | .06250 | .30079 | 1.000 | -.8023 | .9273 |
|  | 3 | .48750 | .30079 | .495 | -.3773 | 1.3523 |
|  | 4 | .20000 | .30079 | .963 | -.6648 | 1.0648 |
| *. The mean difference is significant at the 0.05 level. | | | | | |  |

- 1. **Insulin**  **(After)**

1.6.1. **Data**

3.4 5.4 6.2 4.3 4.4

4.2 4.9 6.4 3.7 6.1

4.6 5.3 5.4 4.9 5.4

3.9 5.7 5.7 3.8 5.7

4.4 4.8 7.1 4.5 4.5

3.3 4.6 7.2 4.1 4.7

3.7 6.1 6.3 4.4 5.3

4.6 6.3 6.6 4.5 4.8

1. **5.4 6.4 4.3 5.1**

| **1.6.2. Statistical analysis** | | | | | | |
| --- | --- | --- | --- | --- | --- | --- |
| **Multiple Comparisons** | | | | | | |
|  | |  |  |  |  |  |
| (I) VAR00003 | (J) VAR00003 | Mean Difference (I-J) | Std. Error | Sig. | 95% Confidence Interval | |
|  |  |  |  |  | Lower Bound | Upper Bound |
| 1 | 2 | -1.37500^*^ | .27660 | .000 | -2.1702 | -.5798 |
|  | 3 | -2.35000^*^ | .27660 | .000 | -3.1452 | -1.5548 |
|  | 4 | -.26250 | .27660 | .876 | -1.0577 | .5327 |
|  | 5 | -1.08750^*^ | .27660 | .003 | -1.8827 | -.2923 |
| 2 | 1 | 1.37500^*^ | .27660 | .000 | .5798 | 2.1702 |
|  | 3 | -.97500^*^ | .27660 | .010 | -1.7702 | -.1798 |
|  | 4 | 1.11250^*^ | .27660 | .003 | .3173 | 1.9077 |
|  | 5 | .28750 | .27660 | .835 | -.5077 | 1.0827 |
| 3 | 1 | 2.35000^*^ | .27660 | .000 | 1.5548 | 3.1452 |
|  | 2 | .97500^*^ | .27660 | .010 | .1798 | 1.7702 |
|  | 4 | 2.08750^*^ | .27660 | .000 | 1.2923 | 2.8827 |
|  | 5 | 1.26250^*^ | .27660 | .001 | .4673 | 2.0577 |
| 4 | 1 | .26250 | .27660 | .876 | -.5327 | 1.0577 |
|  | 2 | -1.11250^*^ | .27660 | .003 | -1.9077 | -.3173 |
|  | 3 | -2.08750^*^ | .27660 | .000 | -2.8827 | -1.2923 |
|  | 5 | -.82500^*^ | .27660 | .039 | -1.6202 | -.0298 |
| 5 | 1 | 1.08750^*^ | .27660 | .003 | .2923 | 1.8827 |
|  | 2 | -.28750 | .27660 | .835 | -1.0827 | .5077 |
|  | 3 | -1.26250^*^ | .27660 | .001 | -2.0577 | -.4673 |
|  | 4 | .82500^*^ | .27660 | .039 | .0298 | 1.6202 |
|  | | | | | |  |

- - 1. **Statistical analysis (Paired Samples Test)**

**Insulin**  **(Before vs. After)**

| **Paired Samples Test^a^** | | | | | | | | | | |
| --- | --- | --- | --- | --- | --- | --- | --- | --- | --- | --- |
| VAR00003 | | | Paired Differences | | | | | t | df | Sig. (2-tailed) |
|  |  |  | Mean | Std. Deviation | Std. Error Mean | 95% Confidence Interval of the Difference | |  |  |  |
|  |  |  |  |  |  | Lower | Upper |  |  |  |
| 1 |  |  | .22500 | .39188 | .13855 | -.10262 | .55262 | 1.624 | 7 | .148 |
| 2 |  |  | .32500 | .61354 | .21692 | -.18793 | .83793 | 1.498 | 7 | .178 |
| 3 |  |  | -1.07500 | 1.10292 | .38994 | -1.99706 | -.15294 | -2.757 | 7 | .028 |
| 4 |  |  | 1.31250 | .73764 | .26079 | .69582 | 1.92918 | 5.033 | 7 | .002 |
| 5 |  |  | .67500 | .77598 | .27435 | .02627 | 1.32373 | 2.460 | 7 | .043 |
