# Supplementary material for: Intracerebroventricular Injection of Alarin Increased Glucose Uptake in Skeletal Muscle of Diabetic Rats
Source: PLoS One. 2015 Oct 6;10(10):e0139327. doi: 10.1371/journal.pone.0139327 (PMC4595443; doi:10.1371/journal.pone.0139327)
Supplement: S2 File — 1.7. Data 1.8. Statistical analysis (DOCX) [file pone.0139327.s002.docx]

1. **Plasma glucose levels**

Fig. 1


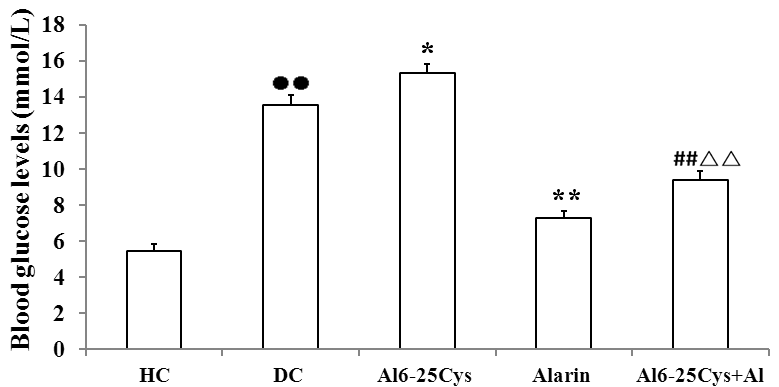


| **2.1. Data**  5.8 | | 13.3 | | 15.3 | | 7.9 | | 8.5 | |  |  |
| --- | --- | --- | --- | --- | --- | --- | --- | --- | --- | --- | --- |
| 5.6 | | 14.3 | | 14.3 | | 4.8 | | 10.2 | |  |  |
| 4.8 | | 13.2 | | 15.2 | | 6.2 | | 9.4 | |  |  |
| 5.7 | | 13.5 | | 15.3 | | 7.3 | | 9.5 | |  |  |
| 6.1 | | 14.2 | | 14.1 | | 8.9 | | 11.9 | |  |  |
| 5.2 | | 12.8 | | 16.6 | | 5.8 | | 10.4 | |  |  |
| 4.6 | | 14.2 | | 16.5 | | 5.5 | | 7.6 | |  |  |
| 4.7 | | 14.5 | | 15.8 | | 8.1 | | 9.8 | |  |  |
|  | |  | |  | |  | |  | |  |  |
| **5.3125** | | **13.75** | | **15.3875** | | **6.8125** | | **9.6625** | |  |  |
|  | |  | |  | |  | |  | |  |  |
| **2.2. Statistical analysis** | |  | |  | |  | |  | |  |  |
|  | | |  | |  | |  | |  | |  |
| (I) VAR00001 | (J) VAR00001 | | Mean Difference (I-J) | | Std. Error | | Sig. | | 95% Confidence Interval | | |
|  |  |  |  |  |  |  |  |  | Lower Bound | | Upper Bound |
| 1 | 2 | | -8.43750^*^ | | .51357 | | .000 | | -9.9140 | | -6.9610 |
|  | 3 | | -10.07500^*^ | | .51357 | | .000 | | -11.5515 | | -8.5985 |
|  | 4 | | -1.50000^*^ | | .51357 | | .045 | | -2.9765 | | -.0235 |
|  | 5 | | -4.35000^*^ | | .51357 | | .000 | | -5.8265 | | -2.8735 |
| 2 | 1 | | 8.43750^*^ | | .51357 | | .000 | | 6.9610 | | 9.9140 |
|  | 3 | | -1.63750^*^ | | .51357 | | .024 | | -3.1140 | | -.1610 |
|  | 4 | | 6.93750^*^ | | .51357 | | .000 | | 5.4610 | | 8.4140 |
|  | 5 | | 4.08750^*^ | | .51357 | | .000 | | 2.6110 | | 5.5640 |
| 3 | 1 | | 10.07500^*^ | | .51357 | | .000 | | 8.5985 | | 11.5515 |
|  | 2 | | 1.63750^*^ | | .51357 | | .024 | | .1610 | | 3.1140 |
|  | 4 | | 8.57500^*^ | | .51357 | | .000 | | 7.0985 | | 10.0515 |
|  | 5 | | 5.72500^*^ | | .51357 | | .000 | | 4.2485 | | 7.2015 |
| 4 | 1 | | 1.50000^*^ | | .51357 | | .045 | | .0235 | | 2.9765 |
|  | 2 | | -6.93750^*^ | | .51357 | | .000 | | -8.4140 | | -5.4610 |
|  | 3 | | -8.57500^*^ | | .51357 | | .000 | | -10.0515 | | -7.0985 |
|  | 5 | | -2.85000^*^ | | .51357 | | .000 | | -4.3265 | | -1.3735 |
| 5 | 1 | | 4.35000^*^ | | .51357 | | .000 | | 2.8735 | | 5.8265 |
|  | 2 | | -4.08750^*^ | | .51357 | | .000 | | -5.5640 | | -2.6110 |
|  | 3 | | -5.72500^*^ | | .51357 | | .000 | | -7.2015 | | -4.2485 |
|  | 4 | | 2.85000^*^ | | .51357 | | .000 | | 1.3735 | | 4.3265 |
| *. The mean difference is significant at the 0.05 level. | | | | | | | | | | |  |
