# Supplementary material for: Intracerebroventricular Injection of Alarin Increased Glucose Uptake in Skeletal Muscle of Diabetic Rats
Source: PLoS One. 2015 Oct 6;10(10):e0139327. doi: 10.1371/journal.pone.0139327 (PMC4595443; doi:10.1371/journal.pone.0139327)
Supplement: S3 File — 3.1. Data 3.2. Statistical analysis (DOCX) [file pone.0139327.s003.docx]

**3. ^3^H-2DG uptake in vivo**

Fig. 2


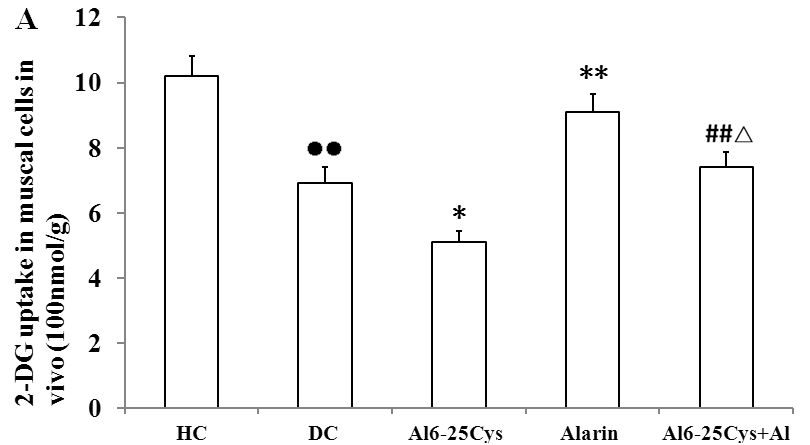


3.1. **Data**

| 1.13 | 0.65 | 0.39 | 0.93 | 0.78 |
| --- | --- | --- | --- | --- |
| 0.93 | 0.82 | 0.63 | 0.85 | 0.76 |
| 1.12 | 0.74 | 0.42 | 0.77 | 0.62 |
| 1.05 | 0.55 | 0.55 | 0.98 | 0.93 |
| 0.82 | 0.61 | 0.64 | 0.95 | 0.69 |
| 1.18 | 0.74 | 0.48 | 0.86 | 0.67 |
| 0.77 | 0.62 | 0.47 | 0.97 | 0.85 |
| 1.14 | 0.84 | 0.48 | 0.94 | 0.61 |
| **10.175** | **6.9625** | **5.075** | **9.0625** | **7.3875** |

**3.2. Statistical analysis**

| **Multiple Comparisons** | | | | | | |
| --- | --- | --- | --- | --- | --- | --- |
|  | |  |  |  |  |  |
| (I) VAR00001 | (J) VAR00001 | Mean Difference (I-J) | Std. Error | Sig. | 95% Confidence Interval | |
|  |  |  |  |  | Lower Bound | Upper Bound |
| 1 | 2 | .32125^*^ | .05573 | .000 | .1610 | .4815 |
|  | 3 | .51000^*^ | .05573 | .000 | .3498 | .6702 |
|  | 4 | .11125 | .05573 | .289 | -.0490 | .2715 |
|  | 5 | .27875^*^ | .05573 | .000 | .1185 | .4390 |
| 2 | 1 | -.32125^*^ | .05573 | .000 | -.4815 | -.1610 |
|  | 3 | .18875^*^ | .05573 | .014 | .0285 | .3490 |
|  | 4 | -.21000^*^ | .05573 | .005 | -.3702 | -.0498 |
|  | 5 | -.04250 | .05573 | .940 | -.2027 | .1177 |
| 3 | 1 | -.51000^*^ | .05573 | .000 | -.6702 | -.3498 |
|  | 2 | -.18875^*^ | .05573 | .014 | -.3490 | -.0285 |
|  | 4 | -.39875^*^ | .05573 | .000 | -.5590 | -.2385 |
|  | 5 | -.23125^*^ | .05573 | .002 | -.3915 | -.0710 |
| 4 | 1 | -.11125 | .05573 | .289 | -.2715 | .0490 |
|  | 2 | .21000^*^ | .05573 | .005 | .0498 | .3702 |
|  | 3 | .39875^*^ | .05573 | .000 | .2385 | .5590 |
|  | 5 | .16750^*^ | .05573 | .037 | .0073 | .3277 |
| 5 | 1 | -.27875^*^ | .05573 | .000 | -.4390 | -.1185 |
|  | 2 | .04250 | .05573 | .940 | -.1177 | .2027 |
|  | 3 | .23125^*^ | .05573 | .002 | .0710 | .3915 |
|  | 4 | -.16750^*^ | .05573 | .037 | -.3277 | -.0073 |
