# Supplementary material for: Intracerebroventricular Injection of Alarin Increased Glucose Uptake in Skeletal Muscle of Diabetic Rats
Source: PLoS One. 2015 Oct 6;10(10):e0139327. doi: 10.1371/journal.pone.0139327 (PMC4595443; doi:10.1371/journal.pone.0139327)
Supplement: S4 File — 1.9. Data 1.10. Statistical analysis (DOCX) [file pone.0139327.s004.docx]

1. **^3^H-2DG uptake in vitro**

Fig.3


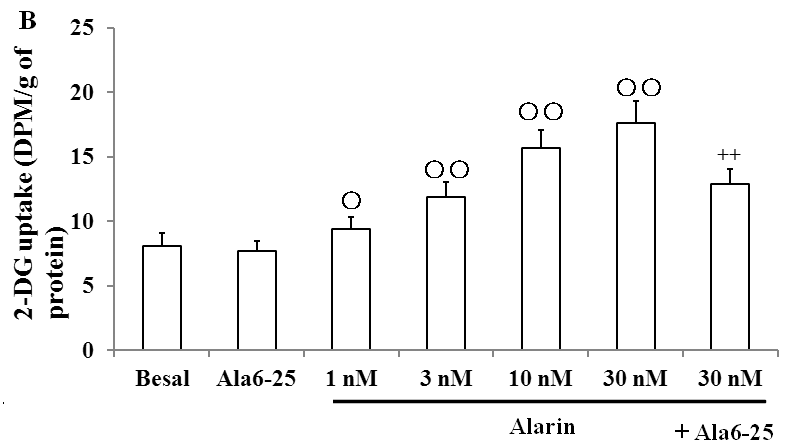


**4.1. Data**

| 7.8 | | 8.2 | | 7.9 | | 13.3 | | 17.5 | | 19.2 | | 13.3 |
| --- | --- | --- | --- | --- | --- | --- | --- | --- | --- | --- | --- | --- |
| 8.2 | | 7.4 | | 10.5 | | 12.5 | | 16.4 | | 17.6 | | 14.1 |
| 8.4 | | 7.8 | | 9.8 | | 11.9 | | 13.4 | | 18.5 | | 12.4 |
| 7.1 | | 6.8 | | 10.4 | | 9.5 | | 16.3 | | 15.7 | | 15.2 |
| 7.5 | | 7.5 | | 8.7 | | 13.2 | | 15.5 | | 16.5 | | 11.8 |
| 9.5 | | 7.4 | | 9.5 | | 12.4 | | 14.8 | | 16.4 | | 10.7 |
| 7.8 | | 6.9 | | 9.7 | | 10.9 | | 15.2 | | 17.8 | | 13.4 |
| 8.3 | | 9.6 | | 8.9 | | 11.1 | | 16.1 | | 19.1 | | 12.5 |
|  | |  | |  | |  | |  | |  | |  |
| **8.075** | | **7.7** | | **9.425** | | **11.85** | | **15.65** | | **17.6** | | **12.925** |
| **4.2. Statistical analysis** | |  | |  | |  | |  | |  | |  |
|  | |  | |  | |  | |  | |  | |  |
|  | |  | |  | |  | |  | |  | |  |
|  | | |  | |  | |  | |  | |  | |
| (I) VAR00003 | (J) VAR00003 | | Mean Difference (I-J) | | Std. Error | | Sig. | | 95% Confidence Interval | | | |
|  |  |  |  |  |  |  |  |  | Lower Bound | | Upper Bound | |
| 1 | 2 | | .37500 | | .56166 | | .994 | | -1.3516 | | 2.1016 | |
|  | 3 | | -1.47500 | | .56166 | | .140 | | -3.2016 | | .2516 | |
|  | 4 | | -3.77500^*^ | | .56166 | | .000 | | -5.5016 | | -2.0484 | |
|  | 5 | | -7.57500^*^ | | .56166 | | .000 | | -9.3016 | | -5.8484 | |
|  | 6 | | -9.52500^*^ | | .56166 | | .000 | | -11.2516 | | -7.7984 | |
|  | 7 | | -4.85000^*^ | | .56166 | | .000 | | -6.5766 | | -3.1234 | |
| 2 | 1 | | -.37500 | | .56166 | | .994 | | -2.1016 | | 1.3516 | |
|  | 3 | | -1.85000^*^ | | .56166 | | .028 | | -3.5766 | | -.1234 | |
|  | 4 | | -4.15000^*^ | | .56166 | | .000 | | -5.8766 | | -2.4234 | |
|  | 5 | | -7.95000^*^ | | .56166 | | .000 | | -9.6766 | | -6.2234 | |
|  | 6 | | -9.90000^*^ | | .56166 | | .000 | | -11.6266 | | -8.1734 | |
|  | 7 | | -5.22500^*^ | | .56166 | | .000 | | -6.9516 | | -3.4984 | |
| 3 | 1 | | 1.47500 | | .56166 | | .140 | | -.2516 | | 3.2016 | |
|  | 2 | | 1.85000^*^ | | .56166 | | .028 | | .1234 | | 3.5766 | |
|  | 4 | | -2.30000^*^ | | .56166 | | .003 | | -4.0266 | | -.5734 | |
|  | 5 | | -6.10000^*^ | | .56166 | | .000 | | -7.8266 | | -4.3734 | |
|  | 6 | | -8.05000^*^ | | .56166 | | .000 | | -9.7766 | | -6.3234 | |
|  | 7 | | -3.37500^*^ | | .56166 | | .000 | | -5.1016 | | -1.6484 | |
| 4 | 1 | | 3.77500^*^ | | .56166 | | .000 | | 2.0484 | | 5.5016 | |
|  | 2 | | 4.15000^*^ | | .56166 | | .000 | | 2.4234 | | 5.8766 | |
|  | 3 | | 2.30000^*^ | | .56166 | | .003 | | .5734 | | 4.0266 | |
|  | 5 | | -3.80000^*^ | | .56166 | | .000 | | -5.5266 | | -2.0734 | |
|  | 6 | | -5.75000^*^ | | .56166 | | .000 | | -7.4766 | | -4.0234 | |
|  | 7 | | -1.07500 | | .56166 | | .481 | | -2.8016 | | .6516 | |
| 5 | 1 | | 7.57500^*^ | | .56166 | | .000 | | 5.8484 | | 9.3016 | |
|  | 2 | | 7.95000^*^ | | .56166 | | .000 | | 6.2234 | | 9.6766 | |
|  | 3 | | 6.10000^*^ | | .56166 | | .000 | | 4.3734 | | 7.8266 | |
|  | 4 | | 3.80000^*^ | | .56166 | | .000 | | 2.0734 | | 5.5266 | |
|  | 6 | | -1.95000^*^ | | .56166 | | .018 | | -3.6766 | | -.2234 | |
|  | 7 | | 2.72500^*^ | | .56166 | | .000 | | .9984 | | 4.4516 | |
| 6 | 1 | | 9.52500^*^ | | .56166 | | .000 | | 7.7984 | | 11.2516 | |
|  | 2 | | 9.90000^*^ | | .56166 | | .000 | | 8.1734 | | 11.6266 | |
|  | 3 | | 8.05000^*^ | | .56166 | | .000 | | 6.3234 | | 9.7766 | |
|  | 4 | | 5.75000^*^ | | .56166 | | .000 | | 4.0234 | | 7.4766 | |
|  | 5 | | 1.95000^*^ | | .56166 | | .018 | | .2234 | | 3.6766 | |
|  | 7 | | 4.67500^*^ | | .56166 | | .000 | | 2.9484 | | 6.4016 | |
| 7 | 1 | | 4.85000^*^ | | .56166 | | .000 | | 3.1234 | | 6.5766 | |
|  | 2 | | 5.22500^*^ | | .56166 | | .000 | | 3.4984 | | 6.9516 | |
|  | 3 | | 3.37500^*^ | | .56166 | | .000 | | 1.6484 | | 5.1016 | |
|  | 4 | | 1.07500 | | .56166 | | .481 | | -.6516 | | 2.8016 | |
|  | 5 | | -2.72500^*^ | | .56166 | | .000 | | -4.4516 | | -.9984 | |
|  | 6 | | -4.67500^*^ | | .56166 | | .000 | | -6.4016 | | -2.9484 | |
