# Supplementary material for: Intracerebroventricular Injection of Alarin Increased Glucose Uptake in Skeletal Muscle of Diabetic Rats
Source: PLoS One. 2015 Oct 6;10(10):e0139327. doi: 10.1371/journal.pone.0139327 (PMC4595443; doi:10.1371/journal.pone.0139327)
Supplement: S5 File — 1.11. Data 1.12. Statistical analysis (DOCX) [file pone.0139327.s005.docx]

**5. Glucose infusion rates**

Fig . 4


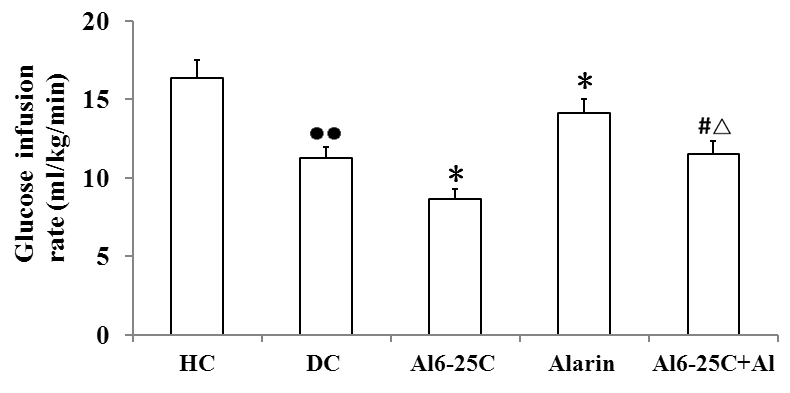


**5.1. Data**

| \| \| 16.4 \| 9.8 \| 6.3 \| 15.3 \| 12.3 \| \| --- \| --- \| --- \| --- \| --- \| \| 17.9 \| 12.3 \| 7.6 \| 12.3 \| 10.6 \| \| 14.5 \| 10.7 \| 7.5 \| 11.1 \| 12.5 \| \| 20.8 \| 9.5 \| 9.7 \| 15.8 \| 11.7 \| \| 16.5 \| 10.6 \| 10.5 \| 15.1 \| 10.5 \| \| 15.2 \| 13.1 \| 9.6 \| 15.3 \| 11.6 \| \| 13.2 \| 11.4 \| 8.3 \| 14.9 \| 13.3 \| \| 16.7 \| 12.8 \| 9.7 \| 13.4 \| 9.7 \| \|  \|  \|  \|  \|  \| \| **16.4** \| **11.275** \| **8.65** \| **14.15** \| **11.525** \| \| **5.2. Statistical analysis** \|  \|  \|  \|  \| \|  \|  \|  \|  \|  \| \| \| \| \| \| \|  \|  \| \|  \|  \| \| \| --- \| --- \| --- \| --- \| --- \| --- \| --- \| --- \| --- \| --- \| --- \| --- \| --- \| --- \| --- \| --- \| --- \| --- \| --- \| --- \| --- \| --- \| --- \| --- \| --- \| --- \| --- \| --- \| --- \| --- \| --- \| --- \| --- \| --- \| --- \| --- \| --- \| --- \| --- \| --- \| --- \| --- \| --- \| --- \| --- \| --- \| --- \| --- \| --- \| --- \| --- \| --- \| --- \| --- \| --- \| --- \| --- \| --- \| --- \| --- \| --- \| --- \| --- \| --- \| --- \| --- \| --- \| --- \| --- \| --- \| --- \| --- \| \| (I) VAR00001 \| (J) VAR00001 \| Mean Difference (I-J) \| Std. Error \| Sig. \| 95% Confidence Interval \| \| \| \| \| \| \| Lower Bound \| \| \| Upper Bound \| \| \| \| 1 \| 2 \| 5.12500^*^ \| .82171 \| .000 \| 2.7625 \| \| \| 7.4875 \| \| \| \| 3 \| 7.75000^*^ \| .82171 \| .000 \| 5.3875 \| \| \| 10.1125 \| \| \| \| 4 \| 2.25000 \| .82171 \| .068 \| -.1125 \| \| \| 4.6125 \| \| \| \| 5 \| 4.87500^*^ \| .82171 \| .000 \| 2.5125 \| \| \| 7.2375 \| \| \| \| 2 \| 1 \| -5.12500^*^ \| .82171 \| .000 \| -7.4875 \| \| \| -2.7625 \| \| \| \| 3 \| 2.62500^*^ \| .82171 \| .023 \| .2625 \| \| \| 4.9875 \| \| \| \| 4 \| -2.87500^*^ \| .82171 \| .011 \| -5.2375 \| \| \| -.5125 \| \| \| \| 5 \| -.25000 \| .82171 \| .998 \| -2.6125 \| \| \| 2.1125 \| \| \| \| 3 \| 1 \| -7.75000^*^ \| .82171 \| .000 \| -10.1125 \| \| \| -5.3875 \| \| \| \| 2 \| -2.62500^*^ \| .82171 \| .023 \| -4.9875 \| \| \| -.2625 \| \| \| \| 4 \| -5.50000^*^ \| .82171 \| .000 \| -7.8625 \| \| \| -3.1375 \| \| \| \| 5 \| -2.87500^*^ \| .82171 \| .011 \| -5.2375 \| \| \| -.5125 \| \| \| \| 4 \| 1 \| -2.25000 \| .82171 \| .068 \| -4.6125 \| \| \| .1125 \| \| \| \| 2 \| 2.87500^*^ \| .82171 \| .011 \| .5125 \| \| \| 5.2375 \| \| \| \| 3 \| 5.50000^*^ \| .82171 \| .000 \| 3.1375 \| \| \| 7.8625 \| \| \| \| 5 \| 2.62500^*^ \| .82171 \| .023 \| .2625 \| \| \| 4.9875 \| \| \| \| 5 \| 1 \| -4.87500^*^ \| .82171 \| .000 \| -7.2375 \| \| \| -2.5125 \| \| \| \| 2 \| .25000 \| .82171 \| .998 \| -2.1125 \| \| \| 2.6125 \| \| \| \| 3 \| 2.87500^*^ \| .82171 \| .011 \| .5125 \| \| \| 5.2375 \| \| \| \| 4 \| -2.62500^*^ \| .82171 \| .023 \| -4.9875 \| \| \| -.2625 \| \| \| \|  \| \| \| \| \| \|  \|  \| \|  \|  \| \| |
| --- | --- | --- | --- | --- | --- | --- | --- | --- | --- | --- | --- | --- | --- | --- | --- | --- | --- | --- | --- | --- | --- | --- | --- | --- | --- | --- | --- | --- | --- | --- | --- | --- | --- | --- | --- | --- | --- | --- | --- | --- | --- | --- | --- | --- | --- | --- | --- | --- | --- | --- | --- | --- | --- | --- | --- | --- | --- | --- | --- | --- | --- | --- | --- | --- | --- | --- | --- | --- | --- | --- | --- | --- | --- | --- | --- | --- | --- | --- | --- | --- | --- | --- | --- | --- | --- | --- | --- | --- | --- | --- | --- | --- | --- | --- | --- | --- | --- | --- | --- | --- | --- | --- | --- | --- | --- | --- | --- | --- | --- | --- | --- | --- | --- | --- | --- | --- | --- | --- | --- | --- | --- | --- | --- | --- | --- | --- | --- | --- | --- | --- | --- | --- | --- | --- | --- | --- | --- | --- | --- | --- | --- | --- | --- | --- | --- | --- | --- | --- | --- | --- | --- | --- | --- | --- | --- | --- | --- | --- | --- | --- | --- | --- | --- | --- | --- | --- | --- | --- | --- | --- | --- | --- | --- | --- | --- | --- | --- | --- | --- | --- | --- | --- | --- | --- | --- | --- | --- | --- | --- | --- | --- | --- | --- | --- | --- | --- | --- | --- | --- | --- | --- | --- | --- | --- | --- | --- | --- | --- | --- | --- | --- | --- | --- | --- | --- | --- | --- | --- | --- | --- | --- | --- | --- | --- | --- | --- | --- | --- | --- | --- | --- | --- | --- | --- | --- | --- | --- | --- | --- | --- | --- | --- | --- | --- | --- | --- | --- | --- | --- | --- | --- | --- | --- | --- | --- | --- | --- | --- | --- | --- | --- | --- | --- | --- | --- | --- | --- | --- | --- | --- | --- | --- | --- | --- | --- | --- | --- | --- | --- | --- | --- | --- | --- | --- | --- | --- | --- | --- | --- | --- | --- | --- | --- | --- | --- | --- | --- | --- | --- | --- | --- | --- | --- | --- | --- | --- |
