# Supplementary material for: Intracerebroventricular Injection of Alarin Increased Glucose Uptake in Skeletal Muscle of Diabetic Rats
Source: PLoS One. 2015 Oct 6;10(10):e0139327. doi: 10.1371/journal.pone.0139327 (PMC4595443; doi:10.1371/journal.pone.0139327)
Supplement: S6 File — 6.1. GLUT4 contents in plasma membranes 6.1.1. Data 6.1.2. Statistical analysis 6.2. GLUT4 contents in total cell membranes 6.2.1. Data 6.2.2. Statistical analysis 6.3. GLUT4 contents in plasma membranes to total cell membranes 6.3.1. Data 6.3.2. Statistical analysis (DOCX) [file pone.0139327.s006.docx]

| **6. GLUT4 contents in membranes of the muscles** | | | | | | |  | |
| --- | --- | --- | --- | --- | --- | --- | --- | --- |
|  |  |  |  |  |  |  |  |  |
|  |  |  |  |  |  |  | |  |

Fig. 5A


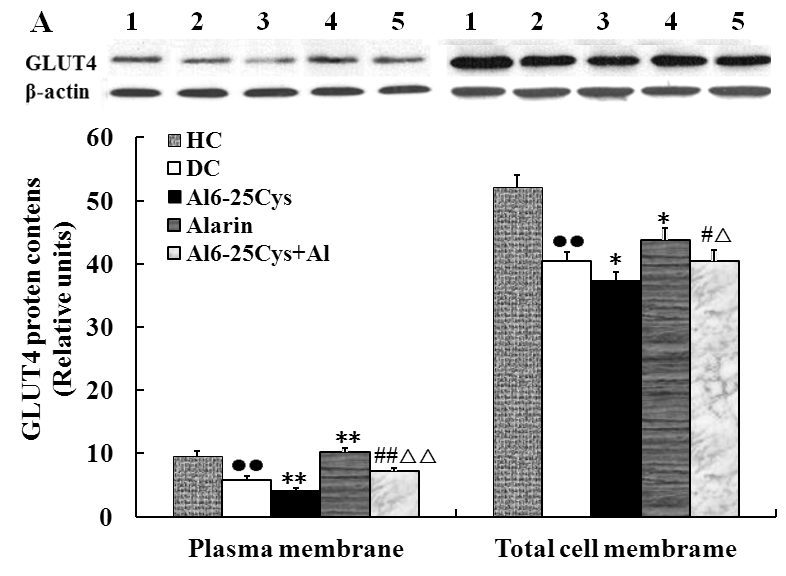


6.1. **GLUT4 contents in plasma membranes**

**6.1.1. Data**

| 10.46 | 4.13 | 3.63 | 11.01 | 7.26 |
| --- | --- | --- | --- | --- |
| 8.57 | 5.65 | 4.65 | 9.86 | 6.54 |
| 9.64 | 4.92 | 3.92 | 10.94 | 6.93 |
| 9.51 | 6.06 | 4.68 | 10.32 | 8.25 |
| 8.91 | 6.11 | 3.86 | 9.78 | 7.06 |
| 9.46 | 5.76 | 3.76 | 9.64 | 7.14 |
| 10.26 | 7.17 | 4.17 | 9.21 | 6.58 |
| 9.34 | 6.43 | 4.23 | 11.17 | 7.46 |
|  |  |  |  |  |
| **9.51875** | **5.77875** | **4.1125** | **10.24125** | **7.1525** |

| **6.1.2. Statistical analysis** | |  | |  | |  | |  |  |  |
| --- | --- | --- | --- | --- | --- | --- | --- | --- | --- | --- |
| (I) VAR00001 | (J) VAR00001 | | Mean Difference (I-J) | | Std. Error | | Sig. | | 95% Confidence Interval | |
|  |  |  |  |  |  |  |  |  | Lower Bound | Upper Bound |
| 1 | 2 | | 3.74000^*^ | | .33452 | | .000 | | 2.7782 | 4.7018 |
|  | 3 | | 5.40625^*^ | | .33452 | | .000 | | 4.4445 | 6.3680 |
|  | 4 | | -.72250 | | .33452 | | .219 | | -1.6843 | .2393 |
|  | 5 | | 2.36625^*^ | | .33452 | | .000 | | 1.4045 | 3.3280 |
| 2 | 1 | | -3.74000^*^ | | .33452 | | .000 | | -4.7018 | -2.7782 |
|  | 3 | | 1.66625^*^ | | .33452 | | .000 | | .7045 | 2.6280 |
|  | 4 | | -4.46250^*^ | | .33452 | | .000 | | -5.4243 | -3.5007 |
|  | 5 | | -1.37375^*^ | | .33452 | | .002 | | -2.3355 | -.4120 |
| 3 | 1 | | -5.40625^*^ | | .33452 | | .000 | | -6.3680 | -4.4445 |
|  | 2 | | -1.66625^*^ | | .33452 | | .000 | | -2.6280 | -.7045 |
|  | 4 | | -6.12875^*^ | | .33452 | | .000 | | -7.0905 | -5.1670 |
|  | 5 | | -3.04000^*^ | | .33452 | | .000 | | -4.0018 | -2.0782 |
| 4 | 1 | | .72250 | | .33452 | | .219 | | -.2393 | 1.6843 |
|  | 2 | | 4.46250^*^ | | .33452 | | .000 | | 3.5007 | 5.4243 |
|  | 3 | | 6.12875^*^ | | .33452 | | .000 | | 5.1670 | 7.0905 |
|  | 5 | | 3.08875^*^ | | .33452 | | .000 | | 2.1270 | 4.0505 |
| 5 | 1 | | -2.36625^*^ | | .33452 | | .000 | | -3.3280 | -1.4045 |
|  | 2 | | 1.37375^*^ | | .33452 | | .002 | | .4120 | 2.3355 |
|  | 3 | | 3.04000^*^ | | .33452 | | .000 | | 2.0782 | 4.0018 |
|  | 4 | | -3.08875^*^ | | .33452 | | .000 | | -4.0505 | -2.1270 |

6.2. **GLUT4 contents in total cell membranes**

**6.2.1. Data**

| 54.32 | 38.59 | 37.25 | 44.83 | 40.83 |
| --- | --- | --- | --- | --- |
| 54.89 | 40.94 | 36.33 | 41.23 | 40.23 |
| 50.22 | 41.01 | 38.62 | 42.42 | 39.42 |
| 49.67 | 42.28 | 37.94 | 44.84 | 41.84 |
| 48.67 | 43.61 | 36.85 | 44.25 | 39.25 |
| 50.34 | 41.94 | 37.18 | 45.27 | 41.27 |
| 52.49 | 35.62 | 36.79 | 42.89 | 39.49 |
| 56.33 | 39.67 | 37.91 | 44.61 | 41.61 |
|  |  |  |  |  |
| **52.11625** | **40.4575** | **37.35875** | **43.7925** | **40.4925** |

**6.2.2. Statistical analysis**

| (I) VAR00001 | (J) VAR00001 | | Mean Difference (I-J) | | Std. Error | Sig. | 95% Confidence Interval | |
| --- | --- | --- | --- | --- | --- | --- | --- | --- |
|  |  |  |  |  |  |  | Lower Bound | Upper Bound |
| 1 | 2 | | 116.58750^*^ | | 9.49431 | .000 | 89.2908 | 143.8842 |
|  | 3 | | 147.57500^*^ | | 9.49431 | .000 | 120.2783 | 174.8717 |
|  | 4 | | 85.73750^*^ | | 9.49431 | .000 | 58.4408 | 113.0342 |
|  | 5 | | 116.23750^*^ | | 9.49431 | .000 | 88.9408 | 143.5342 |
| 2 | 1 | | -116.58750^*^ | | 9.49431 | .000 | -143.8842 | -89.2908 |
|  | 3 | | 30.98750^*^ | | 9.49431 | .019 | 3.6908 | 58.2842 |
|  | 4 | | -30.85000^*^ | | 9.49431 | .020 | -58.1467 | -3.5533 |
|  | 5 | | -.35000 | | 9.49431 | 1.000 | -27.6467 | 26.9467 |
| 3 | 1 | | -147.57500^*^ | | 9.49431 | .000 | -174.8717 | -120.2783 |
|  | 2 | | -30.98750^*^ | | 9.49431 | .019 | -58.2842 | -3.6908 |
|  | 4 | | -61.83750^*^ | | 9.49431 | .000 | -89.1342 | -34.5408 |
|  | 5 | | -31.33750^*^ | | 9.49431 | .018 | -58.6342 | -4.0408 |
| 4 | 1 | | -85.73750^*^ | | 9.49431 | .000 | -113.0342 | -58.4408 |
|  | 2 | | 30.85000^*^ | | 9.49431 | .020 | 3.5533 | 58.1467 |
|  | 3 | | 61.83750^*^ | | 9.49431 | .000 | 34.5408 | 89.1342 |
|  | 5 | | 30.50000^*^ | | 9.49431 | .022 | 3.2033 | 57.7967 |
| 5 | 1 | | -116.23750^*^ | | 9.49431 | .000 | -143.5342 | -88.9408 |
|  | 2 | | .35000 | | 9.49431 | 1.000 | -26.9467 | 27.6467 |
|  | 3 | | 31.33750^*^ | | 9.49431 | .018 | 4.0408 | 58.6342 |
|  | 4 | | -30.50000^*^ | | 9.49431 | .022 | -57.7967 | -3.2033 |
|  | |  | |  |  |  |  |  |

**6.3. GLUT4 contents in plasma membranes to total cell membranes**

Fig. 5B

**6.3.1. Data**
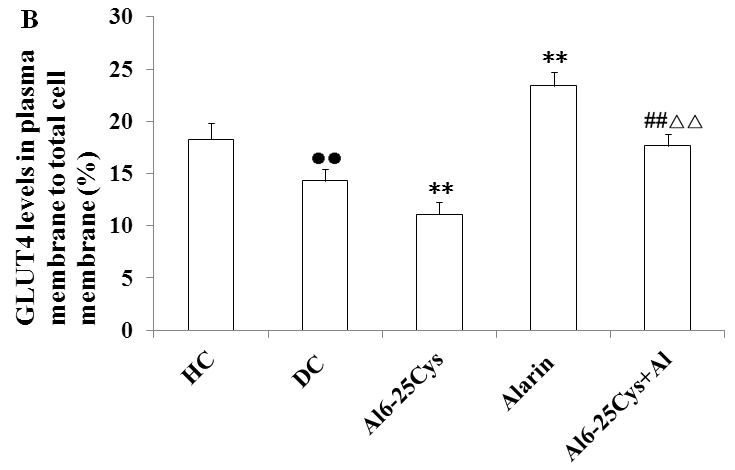


| 20.5 | | 14.6 | | 9.4 | | 23.5 | | 17.6 | |  |  |
| --- | --- | --- | --- | --- | --- | --- | --- | --- | --- | --- | --- |
| 15.9 | | 12.3 | | 10.6 | | 21.9 | | 18.9 | |  |  |
| 19.7 | | 15.1 | | 11.8 | | 23.4 | | 19.1 | |  |  |
| 16.4 | | 14.2 | | 12.5 | | 23.3 | | 18.4 | |  |  |
| 18.5 | | 15.8 | | 14.3 | | 24.6 | | 16.6 | |  |  |
| 16.6 | | 12.6 | | 10.2 | | 23.7 | | 15.4 | |  |  |
| 17.4 | | 13.3 | | 11.6 | | 24.1 | | 17.8 | |  |  |
| 21.1 | | 16.4 | | 8.7 | | 22.5 | | 17.4 | |  |  |
|  | |  | |  | |  | |  | |  |  |
| **6.3.2. Statistical analysis** | |  | |  | |  | |  | |  |  |
|  | |  | |  | |  | |  | |  |  |
| (I) VAR00001 | (J) VAR00001 | | Mean Difference (I-J) | | Std. Error | | Sig. | | 95% Confidence Interval | | |
|  |  |  |  |  |  |  |  |  | Lower Bound | | Upper Bound |
| 1 | 2 | | 4.10000^*^ | | .74350 | | .000 | | 1.9624 | | 6.2376 |
|  | 3 | | 7.25000^*^ | | .74350 | | .000 | | 5.1124 | | 9.3876 |
|  | 4 | | -4.98750^*^ | | .74350 | | .000 | | -7.1251 | | -2.8499 |
|  | 5 | | .73750 | | .74350 | | .857 | | -1.4001 | | 2.8751 |
| 2 | 1 | | -4.10000^*^ | | .74350 | | .000 | | -6.2376 | | -1.9624 |
|  | 3 | | 3.15000^*^ | | .74350 | | .001 | | 1.0124 | | 5.2876 |
|  | 4 | | -9.08750^*^ | | .74350 | | .000 | | -11.2251 | | -6.9499 |
|  | 5 | | -3.36250^*^ | | .74350 | | .001 | | -5.5001 | | -1.2249 |
| 3 | 1 | | -7.25000^*^ | | .74350 | | .000 | | -9.3876 | | -5.1124 |
|  | 2 | | -3.15000^*^ | | .74350 | | .001 | | -5.2876 | | -1.0124 |
|  | 4 | | -12.23750^*^ | | .74350 | | .000 | | -14.3751 | | -10.0999 |
|  | 5 | | -6.51250^*^ | | .74350 | | .000 | | -8.6501 | | -4.3749 |
| 4 | 1 | | 4.98750^*^ | | .74350 | | .000 | | 2.8499 | | 7.1251 |
|  | 2 | | 9.08750^*^ | | .74350 | | .000 | | 6.9499 | | 11.2251 |
|  | 3 | | 12.23750^*^ | | .74350 | | .000 | | 10.0999 | | 14.3751 |
|  | 5 | | 5.72500^*^ | | .74350 | | .000 | | 3.5874 | | 7.8626 |
| 5 | 1 | | -.73750 | | .74350 | | .857 | | -2.8751 | | 1.4001 |
|  | 2 | | 3.36250^*^ | | .74350 | | .001 | | 1.2249 | | 5.5001 |
|  | 3 | | 6.51250^*^ | | .74350 | | .000 | | 4.3749 | | 8.6501 |
|  | 4 | | -5.72500^*^ | | .74350 | | .000 | | -7.8626 | | -3.5874 |
