# Supplementary material for: Intracerebroventricular Injection of Alarin Increased Glucose Uptake in Skeletal Muscle of Diabetic Rats
Source: PLoS One. 2015 Oct 6;10(10):e0139327. doi: 10.1371/journal.pone.0139327 (PMC4595443; doi:10.1371/journal.pone.0139327)
Supplement: S7 File — 7.1. VAMP2 expression levels 7.1.1. Data 7.1.2. Statistical analysis 7.2. GLUT4 mRNA expression levels 6.2.1. Data 6.2.2. Statistical analysis (DOCX) [file pone.0139327.s007.docx]

1. **VAMP2 and GLUT4 mRNA expression levels**

Fig 7


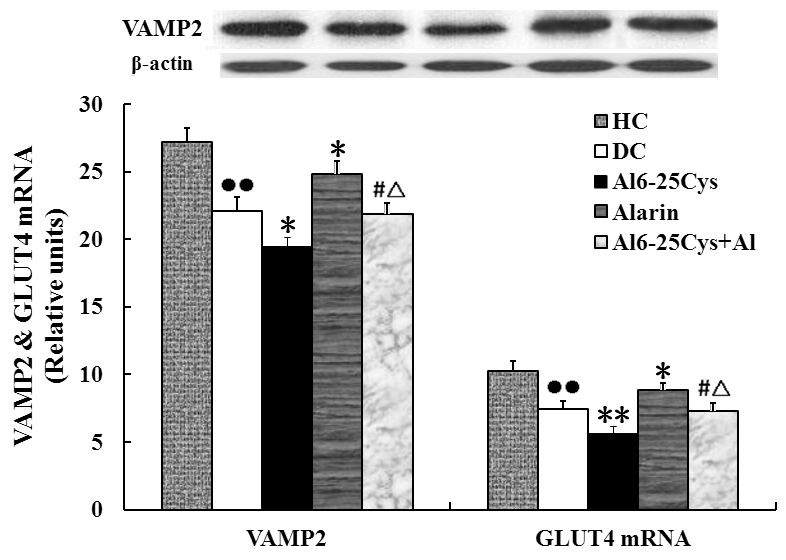


7.1. **VAMP2 expression levels**

| **7.1.1. Data**   \| 26.8 \| 24.4 \| 18.3 \| 25.7 \| 19.3 \| \| --- \| --- \| --- \| --- \| --- \| \| 24.2 \| 21.6 \| 19.3 \| 24.4 \| 24.3 \| \| 29.5 \| 24.7 \| 18.8 \| 26.2 \| 22.8 \| \| 24.8 \| 22.6 \| 19.9 \| 24.8 \| 19.9 \| \| 28.1 \| 20.1 \| 19.8 \| 23.8 \| 21.8 \| \| 27.3 \| 22.4 \| 18.9 \| 23.5 \| 22.9 \| \| 29.8 \| 21.8 \| 20.6 \| 24.9 \| 23.6 \| \| 26.7 \| 19.3 \| 20.1 \| 25.4 \| 20.1 \| \|  \|  \|  \|  \|  \| \| **27.15** \| **22.1125** \| **19.4625** \| **24.8375** \| **21.8375** \| |  |
| --- | --- | --- | --- | --- | --- | --- | --- | --- | --- | --- | --- | --- | --- | --- | --- | --- | --- | --- | --- | --- | --- | --- | --- | --- | --- | --- | --- | --- | --- | --- | --- | --- | --- | --- | --- | --- | --- | --- | --- | --- | --- | --- | --- | --- | --- | --- | --- | --- | --- | --- | --- |

7.1.2. **Statistical analysis**

| (I) VAR00001 | (J) VAR00001 | Mean Difference (I-J) | Std. Error | Sig. | 95% Confidence Interval | |
| --- | --- | --- | --- | --- | --- | --- |
|  |  |  |  |  | Lower Bound | Upper Bound |
| 1 | 2 | 5.03750^*^ | .78022 | .000 | 2.7943 | 7.2807 |
|  | 3 | 7.68750^*^ | .78022 | .000 | 5.4443 | 9.9307 |
|  | 4 | 2.61250^*^ | .78022 | .016 | .3693 | 4.8557 |
|  | 5 | 5.31250^*^ | .78022 | .000 | 3.0693 | 7.5557 |
| 2 | 1 | -5.03750^*^ | .78022 | .000 | -7.2807 | -2.7943 |
|  | 3 | 2.65000^*^ | .78022 | .014 | .4068 | 4.8932 |
|  | 4 | -2.42500^*^ | .78022 | .029 | -4.6682 | -.1818 |
|  | 5 | .27500 | .78022 | .997 | -1.9682 | 2.5182 |
| 3 | 1 | -7.68750^*^ | .78022 | .000 | -9.9307 | -5.4443 |
|  | 2 | -2.65000^*^ | .78022 | .014 | -4.8932 | -.4068 |
|  | 4 | -5.07500^*^ | .78022 | .000 | -7.3182 | -2.8318 |
|  | 5 | -2.37500^*^ | .78022 | .034 | -4.6182 | -.1318 |
| 4 | 1 | -2.61250^*^ | .78022 | .016 | -4.8557 | -.3693 |
|  | 2 | 2.42500^*^ | .78022 | .029 | .1818 | 4.6682 |
|  | 3 | 5.07500^*^ | .78022 | .000 | 2.8318 | 7.3182 |
|  | 5 | 2.70000^*^ | .78022 | .012 | .4568 | 4.9432 |
| 5 | 1 | -5.31250^*^ | .78022 | .000 | -7.5557 | -3.0693 |
|  | 2 | -.27500 | .78022 | .997 | -2.5182 | 1.9682 |
|  | 3 | 2.37500^*^ | .78022 | .034 | .1318 | 4.6182 |
|  | 4 | -2.70000^*^ | .78022 | .012 | -4.9432 | -.4568 |

7.2. **GLUT4 mRNA expression levels**

**7.2.1. Data**

| 8.9 | 6.6 | 5.7 | 8.5 | 6.4 |
| --- | --- | --- | --- | --- |
| 9.4 | 7.2 | 6.3 | 9.4 | 7.2 |
| 12.2 | 8.7 | 5.1 | 8.1 | 7.6 |
| 11.6 | 6.5 | 5.5 | 9.5 | 6.3 |
| 10.4 | 6.6 | 5.2 | 9.2 | 8.2 |
| 9.4 | 7.6 | 5.9 | 7.8 | 7.1 |
| 8.1 | 8.7 | 5.4 | 9.6 | 7.4 |
| 12.3 | 7.4 | 6.1 | 8.6 | 8.1 |
|  |  |  |  |  |
| **10.2875** | **7.4125** | **5.65** | **8.8375** | **7.2875** |

**7.2.2. Statistical analysis**

| (I) VAR00001 | (J) VAR00001 | Mean Difference (I-J) | Std. Error | Sig. | 95% Confidence Interval | |
| --- | --- | --- | --- | --- | --- | --- |
|  |  |  |  |  | Lower Bound | Upper Bound |
| 1 | 2 | 2.87500^*^ | .47828 | .000 | 1.4999 | 4.2501 |
|  | 3 | 4.63750^*^ | .47828 | .000 | 3.2624 | 6.0126 |
|  | 4 | 1.45000^*^ | .47828 | .035 | .0749 | 2.8251 |
|  | 5 | 3.00000^*^ | .47828 | .000 | 1.6249 | 4.3751 |
| 2 | 1 | -2.87500^*^ | .47828 | .000 | -4.2501 | -1.4999 |
|  | 3 | 1.76250^*^ | .47828 | .006 | .3874 | 3.1376 |
|  | 4 | -1.42500^*^ | .47828 | .039 | -2.8001 | -.0499 |
|  | 5 | .12500 | .47828 | .999 | -1.2501 | 1.5001 |
| 3 | 1 | -4.63750^*^ | .47828 | .000 | -6.0126 | -3.2624 |
|  | 2 | -1.76250^*^ | .47828 | .006 | -3.1376 | -.3874 |
|  | 4 | -3.18750^*^ | .47828 | .000 | -4.5626 | -1.8124 |
|  | 5 | -1.63750^*^ | .47828 | .013 | -3.0126 | -.2624 |
| 4 | 1 | -1.45000^*^ | .47828 | .035 | -2.8251 | -.0749 |
|  | 2 | 1.42500^*^ | .47828 | .039 | .0499 | 2.8001 |
|  | 3 | 3.18750^*^ | .47828 | .000 | 1.8124 | 4.5626 |
|  | 5 | 1.55000^*^ | .47828 | .021 | .1749 | 2.9251 |
| 5 | 1 | -3.00000^*^ | .47828 | .000 | -4.3751 | -1.6249 |
|  | 2 | -.12500 | .47828 | .999 | -1.5001 | 1.2501 |
|  | 3 | 1.63750^*^ | .47828 | .013 | .2624 | 3.0126 |
|  | 4 | -1.55000^*^ | .47828 | .021 | -2.9251 | -.1749 |
