# Supplementary material for: Intracerebroventricular Injection of Alarin Increased Glucose Uptake in Skeletal Muscle of Diabetic Rats
Source: PLoS One. 2015 Oct 6;10(10):e0139327. doi: 10.1371/journal.pone.0139327 (PMC4595443; doi:10.1371/journal.pone.0139327)
Supplement: S8 File — 8.1. pAktThr308 levels 8.1.1. Data 8.1.2. Statistical analysis 8.2. pAktSer473 levels 8.2.1. Data 8.2.2. Statistical analysis 8.3. Total Akt levels 8.3.1. Data 8.3.2. Statistical analysis (DOCX) [file pone.0139327.s008.docx]

1. **pAkt levels**

Fig. 8


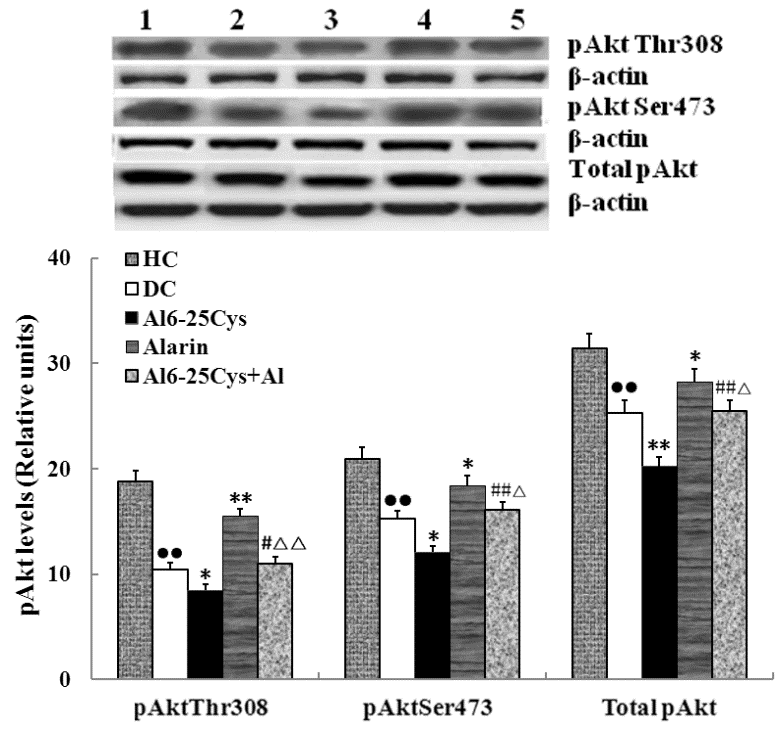


| **8.1.** **pAkt^Thr308^ levels**  **8.1.1. Data** |  |  |  |  |
| --- | --- | --- | --- | --- |
|  |  |  |  |  |
| 21.3 | 7.9 | 7.9 | 14.5 | 10.9 |
| 17.1 | 9.7 | 8.7 | 14.8 | 11.8 |
| 18.6 | 10.2 | 7.2 | 16.5 | 10.3 |
| 18.2 | 10.9 | 9.9 | 13.6 | 9.4 |
| 19.4 | 12.6 | 8.6 | 15.6 | 9.6 |
| 17.8 | 12.3 | 7.3 | 17.1 | 9.1 |
| 19.3 | 8.8 | 8.8 | 16.2 | 10.3 |
| 18.8 | 11.3 | 9.3 | 15.8 | 11.3 |
|  |  |  |  |  |
| 18.8 | 10.5 | 8.5 | 15.5 | 10.34 |
|  |  |  |  |  |

**8.1.2. Statistical analysis**

| (I) VAR00001 | (J) VAR00001 | Mean Difference (I-J) | Std. Error | Sig. | 95% Confidence Interval | |
| --- | --- | --- | --- | --- | --- | --- |
|  |  |  |  |  | Lower Bound | Upper Bound |
| 1 | 2 | 8.35000^*^ | .62045 | .000 | 6.5662 | 10.1338 |
|  | 3 | 10.35000^*^ | .62045 | .000 | 8.5662 | 12.1338 |
|  | 4 | 3.30000^*^ | .62045 | .000 | 1.5162 | 5.0838 |
|  | 5 | 8.27500^*^ | .62045 | .000 | 6.4912 | 10.0588 |
| 2 | 1 | -8.35000^*^ | .62045 | .000 | -10.1338 | -6.5662 |
|  | 3 | 2.00000^*^ | .62045 | .022 | .2162 | 3.7838 |
|  | 4 | -5.05000^*^ | .62045 | .000 | -6.8338 | -3.2662 |
|  | 5 | -.07500 | .62045 | 1.000 | -1.8588 | 1.7088 |
| 3 | 1 | -10.35000^*^ | .62045 | .000 | -12.1338 | -8.5662 |
|  | 2 | -2.00000^*^ | .62045 | .022 | -3.7838 | -.2162 |
|  | 4 | -7.05000^*^ | .62045 | .000 | -8.8338 | -5.2662 |
|  | 5 | -2.07500^*^ | .62045 | .016 | -3.8588 | -.2912 |
| 4 | 1 | -3.30000^*^ | .62045 | .000 | -5.0838 | -1.5162 |
|  | 2 | 5.05000^*^ | .62045 | .000 | 3.2662 | 6.8338 |
|  | 3 | 7.05000^*^ | .62045 | .000 | 5.2662 | 8.8338 |
|  | 5 | 4.97500^*^ | .62045 | .000 | 3.1912 | 6.7588 |
| 5 | 1 | -8.27500^*^ | .62045 | .000 | -10.0588 | -6.4912 |
|  | 2 | .07500 | .62045 | 1.000 | -1.7088 | 1.8588 |
|  | 3 | 2.07500^*^ | .62045 | .016 | .2912 | 3.8588 |
|  | 4 | -4.97500^*^ | .62045 | .000 | -6.7588 | -3.1912 |

| **8.2. pAkt^Ser473^ levels**  **8.2.1. Data**   \| 21.3 \| 15.7 \| 9.2 \| 17.9 \| 18.2 \| \| --- \| --- \| --- \| --- \| --- \| \| 23.1 \| 14.4 \| 16.4 \| 17.8 \| 16.8 \| \| 18.6 \| 12.9 \| 10.5 \| 19.9 \| 15.6 \| \| 24.2 \| 18.6 \| 16.3 \| 19.6 \| 13.3 \| \| 19.4 \| 14.6 \| 11.2 \| 18.9 \| 13.6 \| \| 22.8 \| 16.5 \| 12.8 \| 16.6 \| 15.9 \| \| 19.3 \| 15.5 \| 12.1 \| 21.1 \| 17.5 \| \| 18.8 \| 14.1 \| 8.1 \| 19.5 \| 15.5 \| \|  \|  \|  \|  \|  \| \| **20.9** \| **15.3** \| **12.1** \| **18.9** \| **15.8** \| |  |  |  |  |
| --- | --- | --- | --- | --- | --- | --- | --- | --- | --- | --- | --- | --- | --- | --- | --- | --- | --- | --- | --- | --- | --- | --- | --- | --- | --- | --- | --- | --- | --- | --- | --- | --- | --- | --- | --- | --- | --- | --- | --- | --- | --- | --- | --- | --- | --- | --- | --- | --- | --- | --- | --- | --- | --- | --- |

**8.2.2**. **Statistical analysis**

| (I) VAR00001 | (J) VAR00001 | Mean Difference (I-J) | Std. Error | Sig. | 95% Confidence Interval | |
| --- | --- | --- | --- | --- | --- | --- |
|  |  |  |  |  | Lower Bound | Upper Bound |
| 1 | 2 | 5.65000^*^ | 1.05048 | .000 | 2.6298 | 8.6702 |
|  | 3 | 8.86250^*^ | 1.05048 | .000 | 5.8423 | 11.8827 |
|  | 4 | 2.02500 | 1.05048 | .322 | -.9952 | 5.0452 |
|  | 5 | 5.13750^*^ | 1.05048 | .000 | 2.1173 | 8.1577 |
| 2 | 1 | -5.65000^*^ | 1.05048 | .000 | -8.6702 | -2.6298 |
|  | 3 | 3.21250^*^ | 1.05048 | .032 | .1923 | 6.2327 |
|  | 4 | -3.62500^*^ | 1.05048 | .012 | -6.6452 | -.6048 |
|  | 5 | -.51250 | 1.05048 | .988 | -3.5327 | 2.5077 |
| 3 | 1 | -8.86250^*^ | 1.05048 | .000 | -11.8827 | -5.8423 |
|  | 2 | -3.21250^*^ | 1.05048 | .032 | -6.2327 | -.1923 |
|  | 4 | -6.83750^*^ | 1.05048 | .000 | -9.8577 | -3.8173 |
|  | 5 | -3.72500^*^ | 1.05048 | .009 | -6.7452 | -.7048 |
| 4 | 1 | -2.02500 | 1.05048 | .322 | -5.0452 | .9952 |
|  | 2 | 3.62500^*^ | 1.05048 | .012 | .6048 | 6.6452 |
|  | 3 | 6.83750^*^ | 1.05048 | .000 | 3.8173 | 9.8577 |
|  | 5 | 3.11250^*^ | 1.05048 | .041 | .0923 | 6.1327 |
| 5 | 1 | -5.13750^*^ | 1.05048 | .000 | -8.1577 | -2.1173 |
|  | 2 | .51250 | 1.05048 | .988 | -2.5077 | 3.5327 |
|  | 3 | 3.72500^*^ | 1.05048 | .009 | .7048 | 6.7452 |
|  | 4 | -3.11250^*^ | 1.05048 | .041 | -6.1327 | -.0923 |

**8.3. Total Akt levels**

**8.3.1. Data**

| 30.8 | 26.1 | 18.6 | 26.8 | 26.3 |
| --- | --- | --- | --- | --- |
| 32.4 | 24.3 | 17.2 | 29.1 | 25.6 |
| 29.7 | 27.1 | 20.4 | 29.7 | 27.1 |
| 28.9 | 22.5 | 21.6 | 28.5 | 26.5 |
| 33.7 | 24.6 | 19.3 | 27.6 | 23.7 |
| 31.5 | 26.2 | 20.6 | 26.4 | 24.7 |
| 30.8 | 27.4 | 21.1 | 27.8 | 24.9 |
| 33.2 | 24.5 | 23.1 | 27.7 | 25.2 |
| **31.4** | **25.3** | **20.2375** | **28.3** | **25.5** |

**8.3.2. Statistical analysis**

| 1 | 2 | 6.03750^*^ | .75286 | .000 | 3.8730 | 8.2020 |
| --- | --- | --- | --- | --- | --- | --- |
|  | 3 | 11.13750^*^ | .75286 | .000 | 8.9730 | 13.3020 |
|  | 4 | 3.42500^*^ | .75286 | .001 | 1.2605 | 5.5895 |
|  | 5 | 5.87500^*^ | .75286 | .000 | 3.7105 | 8.0395 |
| 2 | 1 | -6.03750^*^ | .75286 | .000 | -8.2020 | -3.8730 |
|  | 3 | 5.10000^*^ | .75286 | .000 | 2.9355 | 7.2645 |
|  | 4 | -2.61250^*^ | .75286 | .011 | -4.7770 | -.4480 |
|  | 5 | -.16250 | .75286 | .999 | -2.3270 | 2.0020 |
| 3 | 1 | -11.13750^*^ | .75286 | .000 | -13.3020 | -8.9730 |
|  | 2 | -5.10000^*^ | .75286 | .000 | -7.2645 | -2.9355 |
|  | 4 | -7.71250^*^ | .75286 | .000 | -9.8770 | -5.5480 |
|  | 5 | -5.26250^*^ | .75286 | .000 | -7.4270 | -3.0980 |
| 4 | 1 | -3.42500^*^ | .75286 | .001 | -5.5895 | -1.2605 |
|  | 2 | 2.61250^*^ | .75286 | .011 | .4480 | 4.7770 |
|  | 3 | 7.71250^*^ | .75286 | .000 | 5.5480 | 9.8770 |
|  | 5 | 2.45000^*^ | .75286 | .020 | .2855 | 4.6145 |
| 5 | 1 | -5.87500^*^ | .75286 | .000 | -8.0395 | -3.7105 |
|  | 2 | .16250 | .75286 | .999 | -2.0020 | 2.3270 |
|  | 3 | 5.26250^*^ | .75286 | .000 | 3.0980 | 7.4270 |
|  | 4 | -2.45000^*^ | .75286 | .020 | -4.6145 | -.2855 |
